# Bi-stability in cooperative transport by ants in the presence of obstacles

**DOI:** 10.1371/journal.pcbi.1006068

**Published:** 2018-05-10

**Authors:** Jonathan E. Ron, Itai Pinkoviezky, Ehud Fonio, Ofer Feinerman, Nir S. Gov

**Affiliations:** 1 Department of Chemical and Biological Physics, Weizmann Institute, Rehovot, Israel; 2 Department of Biology, Emory University, Atlanta, Georgia, USA; 3 Department of Physics of Complex Systems, Weizmann Institute, Rehovot, Israel; University of Pittsburgh, UNITED STATES

## Abstract

To cooperatively carry large food items to the nest, individual ants conform their efforts and coordinate their motion. Throughout this expedition, collective motion is driven both by internal interactions between the carrying ants and a response to newly arrived informed ants that orient the cargo towards the nest. During the transport process, the carrying group must overcome obstacles that block their path to the nest. Here, we investigate the dynamics of cooperative transport, when the motion of the ants is frustrated by a linear obstacle that obstructs the motion of the cargo. The obstacle contains a narrow opening that serves as the only available passage to the nest, and through which single ants can pass but not with the cargo. We provide an analytical model for the ant-cargo system in the constrained environment that predicts a bi-stable dynamic behavior between an oscillatory mode of motion along the obstacle and a convergent mode of motion near the opening. Using both experiments and simulations, we show how for small cargo sizes, the system exhibits spontaneous transitions between these two modes of motion due to fluctuations in the applied force on the cargo. The bi-stability provides two possible problem solving strategies for overcoming the obstacle, either by attempting to pass through the opening, or take large excursions to circumvent the obstacle.

## Author summary

Among animal groups, ants hold what may perhaps be the richest repertoire of collective behavior such as trail formation, nest excavation and food dissemination. Of the most intriguing of these behaviors is cooperative food transport, where many ants carry items that individuals cannot move. Using experiments and theory, we study cooperative transport when the motion is frustrated by an obstacle which contains a single narrow opening that leads to the nest. We find that the group exhibits two co-existing modes of motion that allow exploration of possible routes to overcome the obstacle: Either dwelling near the opening and attempting to pass the cargo through, or performing large excursions that can lead to obstacle circumvention. Previous studies have found that co-existing collective dynamic modes emerge when animal groups interact with constraints, however the origin of the phenomena remains unknown. Here, we provide a detailed theoretical explanation of the source of bi-stability and show how stochastic processes drive the transitions between the two dynamical modes.

## Introduction

Many living groups exhibit collective modes of motion [1]. Among these, groups such as cell clusters [2, 3], locust [4] and fish [5], have been found to display spontaneous transitions between co-existing collective dynamical phases. Among these collective phases are disordered modes of motion, in which the group swarms whilst remaining cohesive, and ordered modes of motion, where the individuals orient along a single polarized direction, or rotate around the group center of mass. The spontaneous transitions between these collective modes of motion have been attributed to noise [2, 4], or interactions with external constraints [3, 5]. However, there is currently no theoretical description that defines the necessary conditions for the emergence of co-existing dynamical phases, or a precise mechanism that explains the transitions between the different modes of motion. In this study, we investigate dynamical bi-stability, and its theoretical underpinnings during cooperative transport by a group of ants.

Cooperative transport by ants, also known as group retrieval, is the process by which individual ants join efforts to retrieve large items of food [6–10]. Cooperative transport is known to exist in at least forty different ant species [11–20]. Among these species, the longhorn crazy ants *Paratrechina longicornis* are well known for displaying highly coordinated retrieval, of items, that can reach orders of magnitude larger than their own size and weight [13, 21, 22]. After a recruitment phase [22], the longhorn crazy ants lift the load above the surface to reduce friction, and pull towards chemical depositions they leave [22], which mark the pheromone scent trail that leads to the nest [23].

**Fig 1:**
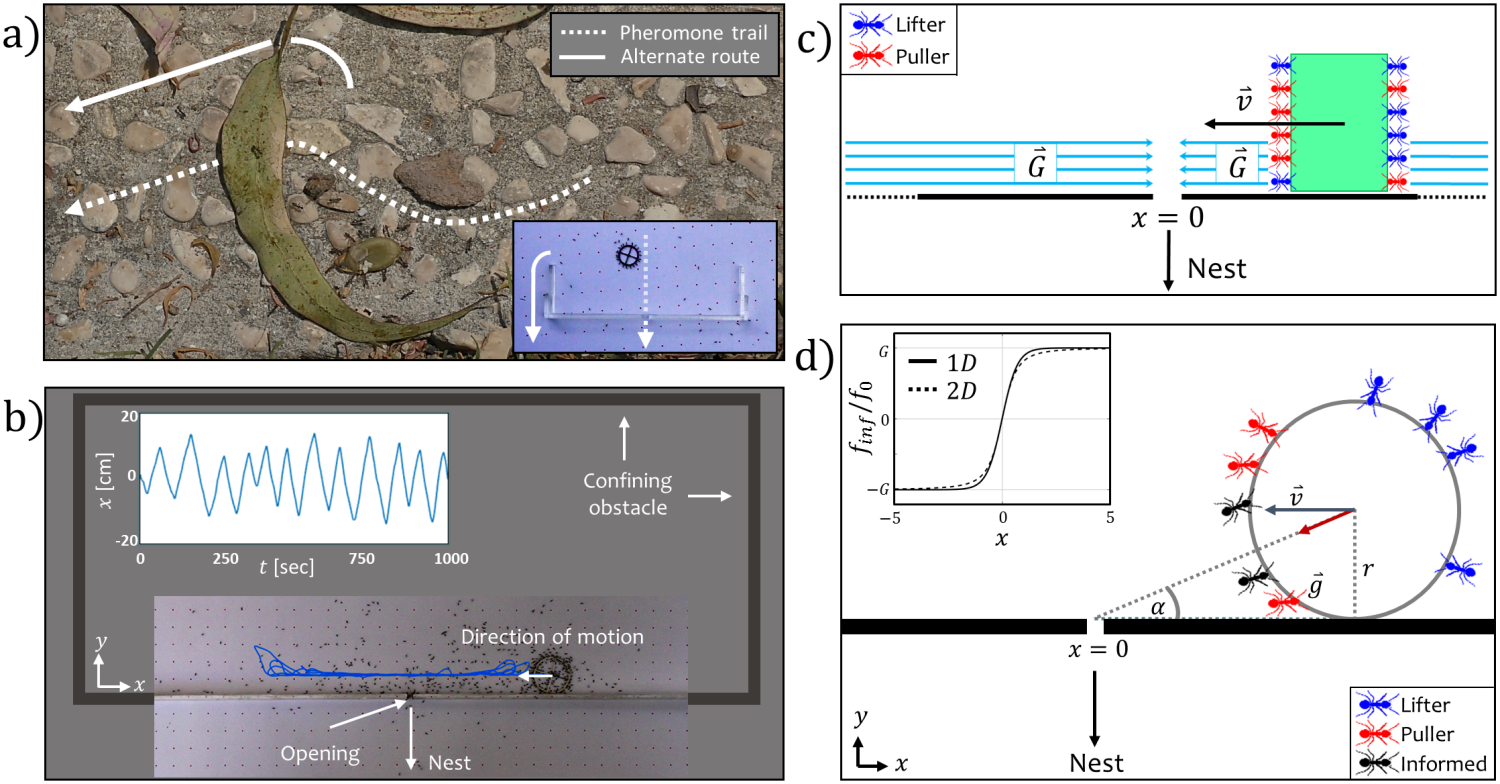
The simplified model. (A) Ants encounter an obstacle during cooperative transport. Dashed line indicates the pheromone scent trail that passes beneath the leaf. Solid line indicates an alternative route. The leaf obstructs the direct route to the nest, and the cargo can not be passed along the original scent trail. Bottom left inset shows a previously studied obstacle [21, 22, 24] that resemble this natural scenario. (B) Ants carrying a circular cargo along a confined linear obstacle with an opening. Blue line is a 16 minute trajectory. The sketched part of the obstacle indicates the true proportions of the enclosed frame. (C) An illustration of the simplified one dimensional model. *G* is the number of informed ants, that are modeled as a restoring force and *v* is the velocity the cargo. Ant color index: Blue—lifter, red—puller, informed ants are represented by the force $\vec{G}$. (D) The restoring force due to the informed ants in the 2D case. Left inset compares between the projection of the restoring force for a two dimensional cargo along the *x* axis, and the restoring force of the continuous function we used in the simplified model for *ϵ* = 1 and *r* = 1 (Eq (9)). The restoring force is normalized by *f*_0_. Ant color index: Blue—lifter, red—puller, black—informed.

Cooperative transport by *P. longicornis* ants was shown to exhibit a rich variety of collective modes which range from random motions to ballistic movement [21], that is either directed towards the nest [21] or exhibits oscillatory modes [24] and direction changes when the motion was externally constrained [22, 25] by semi-natural obstacles (Fig 1A). In addition, cooperative transport by this species has allowed for a comprehensive theoretical description [21, 24]. The existence of multiple modes of motion, along with the theoretical understanding, suggest the potential of this system for investigating the origins and dynamics of co-existing collective modes.

To test this possibility, we have used a simple experimental system, in which the ants interact with a rigid obstacle. Under this constraint, the paths to the nest are blocked from all directions, except for a single narrow opening, that allows passage for single ants, but not for the cargo (Fig 1B and Methods section). In the presence of such an obstacle, ants that accompany the transport of a large cargo were observed to locally mark pheromone trails that lead from the cargo to the narrow opening [22]. Even though these trails lead to a dead end (the cargo can not fit through the opening), the markings serve as the only available source of information, as new ants that join the carrying effort pull and direct the cargo towards the narrow opening [22]. Furthermore, the simple geometry of the obstacle makes the motion of the ant group amenable to analytic theoretical description, which allows us to expose the conditions for bi-stability, and the mechanisms governing the transitions between the co-existing dynamical modes of motion.

Here, we present a theory of cooperative transport by ants near rigid obstacles, which predicts bi-stability between two dynamical modes of motion: a convergent mode of motion that keeps the cargo near the opening, and large quasi periodic excursions that can circumvent an obstacle. We demonstrate how noise in the discrete decision making process of the ants induces mechanical fluctuations that can stochastically switch the group between these two modes. The predictions of the model are then verified in experiments, where ants carrying cargoes with the size of natural prey [21] exhibited spontaneous transitions between the bi-stable collective dynamical modes. In addition to the agreement between observations and simulations, our analytical model allows us to explain the conditions for bi-stability, and propose a physical mechanism for how groups of ants overcome obstacles when carrying food to the nest.

## Results

### The model

To describe the motion of a rigid cargo along an obstacle, we use the model of force driven coupling between ants [21, 24]. In this theory, the carrying ants can either pull the cargo along their body axis, or lift the cargo to reduce its friction with the surface. Based on previous observations [21], we divide the carrying ants in our model into two groups:

**• Uninformed ants:** Carrying ants with no recollection regarding the location of the pheromone trail. These ants switch between pulling and lifting roles according to the forces that they sense, and can detach at a constant average rate [21].

**• Informed ants:** Newly attached ants that arrive with spatial information about the direction of the pheromone trail. These ants transfer the information to the group by pulling the cargo towards the trail, until they lose their directionality at a constant average rate and become uninformed [21].

In this model, the uninformed carrying ants can not sense the pheromone trail, and the navigation relies on the pulling forces of the informed ants that steer the group towards the pheromone trail.

The uninformed ants role switch between pulling and lifting at rates given by

$$\begin{array}{c} r_{l\to p}=k_{c}exp\left(+\frac{{\vec{f}}_{tot}\cdot {\vec{p}}_{i}}{F_{ind}}\right) \end{array}$$ (1)

$$\begin{array}{c} r_{p\to l}=k_{c}exp\left(-\frac{{\vec{f}}_{tot}\cdot {\vec{p}}_{i}}{F_{ind}}\right) \end{array}$$ (2)

where *p*/*l* note the pullers/lifters, *k*_*c*_ is the basal role switching rate, ${\vec{f}}_{tot}$ is the total pulling force applied by the puller ants and ${\vec{p}}_{i}$ is the body axis unit vector of an ant labeled by index *i* along the cargo. The scalar multiplication ${\vec{f}}_{tot}\cdot {\vec{p}}_{i}$ in Eqs (1) and (2) imply that each individual ant instantaneously senses the mechanical forces applied by all other ants. When the direction in which an ant can pull, ${\vec{p}}_{i}$, is aligned with ${\vec{f}}_{tot}$, Eqs (1) and (2) provide a low (high) rate to switch from lifter to puller (puller to lifter). This implies that ants in the front (back) of the cargo will tend to be pullers (lifters), and gives rise to coordinated pulling and cooperative transport. When the cargo changes direction of motion, the ants switch their roles according to Eqs (1) and (2), such that lifters that used to be in the back become predominantly pullers in the new front.

The individuality parameter *F*_*ind*_ in Eqs (1) and (2) plays the role of the temperature in the Ising model [26], where the pullers and lifters are analogue to the binary spin states. The individuality parameter is an ant-level trait, which defines the threshold of forces above which single ants respond to the group [21]. For low *F*_*ind*_ the ants conform and align their forces, while for high *F*_*ind*_ the ants ignore the forces applied by the rest of the group. We treat *F*_*ind*_ as a constant average value among all ants, while it may vary between individuals (previous calculations show that such a variability does not lead to any qualitative change in the collective behavior [21]).

Due to the roughly one-dimensional nature of the motion along the obstacle (Fig 1A), we consider a cargo that is confined to move on an infinite line along the *x* axis. The cargo has *n*_*tot*_ binding sites that are equally distributed between the front and the back sides of the cargo, that face the ±*x* direction respectively. We neglect the processes of attachment/detachment from the cargo, and consider the cargo to be fully occupied by a fixed population of uninformed ants that decide on their role at rates given by Eqs (1) and (2). Note that the cargo has no dimension of length (point particle).

In the presence of an opening located at *x* = 0, the cargo force balance equation accounts the sum of all the pulling and lifting forces applied to the cargo

$$\begin{array}{c} f_{tot}=f_{0}\left(\underset{\text{uninformed}\;\text{pullers}}{\underset{︸}{n_{p}^{front}-n_{p}^{back}}}-\underset{\text{informed}\;\text{pullers}}{\underset{︸}{G\cdot sign(x)}}-\underset{\text{uninformed}\;\text{lifters}}{\underset{︸}{h(n_{l})\;/}}\right) \end{array}$$ (3)

where *f*_0_ is the magnitude applied by a single ant, and is equal to all ants. The first term on the right hand side of Eq (3) describes a tug of war between the uninformed pullers *n*_*p*_ on both sides of the cargo, while the second term describes the pulling force of the informed ants, where *G* is a fixed number of informed pullers that direct the cargo towards the opening at *x* = 0. The function *h*(*n*_*l*_) describes the role of the *n*_*l*_ lifter ants, that reduce the friction of the cargo with the surface (S1 Appendix). Throughout the model, we consider the cargo to be fully lifted above the surface, such that the friction force is neglected.

The total force (3) can be converted to velocity by using the linear relation

$$\begin{array}{c} f_{tot}=\gamma v \end{array}$$ (4)

where *γ* is the mechanical cargo response coefficient [21]. We assume a linear relation since the cargo is held steady throughout the motion by the ants, which have very high friction with the surface, thus keeping the motion in a highly damped non-inertial regime.

The acceleration of the cargo is therefore obtained by plugging Eq (4) into Eq (3) and taking the time derivative of *v*

$$\begin{array}{c} \dot{v}=\frac{f_{0}}{\gamma }\left(\frac{d}{dt}\left(n_{p}^{front}-n_{p}^{back}\right)-G\frac{d}{dx}sign\left(x\right)\frac{dx}{dt}\right) \end{array}$$ (5)

By considering the discrete stochastic role switching dynamics of the uninformed ants (S1 Appendix), we obtain the mean drift velocity of the cargo, as a result of the tug of war

$$\begin{array}{c} \frac{d}{dt}\left(n_{p}^{front}-n_{p}^{back}\right)=\frac{k_{c}f_{0}}{\gamma }\left[\left(n_{l}^{front}+n_{p}^{back}\right)exp\left(\frac{f_{tot}}{F_{ind}}\right)-\left(n_{p}^{front}+n_{l}^{back}\right)exp\left(-\frac{f_{tot}}{F_{ind}}\right)\right] \end{array}$$ (6)

Under our assumption of a fully occupied cargo, Eq (6) can be written in terms of the total number of ants *n*_*tot*_, the mean velocity *v*, and the magnitude of the restoring force *G* (Eqs.S22-S26 in S1 Appendix).

The final form of the equations of motion, that represent the deterministic evolution of the average position and average velocity of the cargo are given by

$$\begin{array}{c} \dot{x}=v \end{array}$$ (7)

$$\begin{array}{c} \dot{v}=q\left(x,v\right) \end{array}$$ (8)

$$\begin{array}{c} q\left(x,v\right)=k_{c}\left[n\cdot sinh\left(\frac{v}{f_{ind}}\right)-2\left(v+g\cdot tanh\left(\frac{x}{\epsilon }\right)\right)cosh\left(\frac{v}{f_{ind}}\right)\right]-v\frac{g}{\epsilon }sech^{2}\left(\frac{x}{\epsilon }\right) \end{array}$$ (9)

where $n=\frac{f_{0}n_{tot}}{\gamma }$, $f_{ind}=\frac{F_{ind}}{\gamma }$ and $g=\frac{f_{0}G}{\gamma }$. The approximation *sign*(*x*) ≈ *tanh*(*x*/*ϵ*) is used to avoid discontinuities when passing over *x* = 0.

The parameter *ϵ* can be related to the cargo radius *r*, as by considering the motion of the cargo along the obstacle in a two dimensional plane, the projection of the restoring force on the *x* axis yields a similar results to our approximation when *ϵ* ∼ *r* (Fig 1C).

### Bifurcation analysis

**Fig 2:**
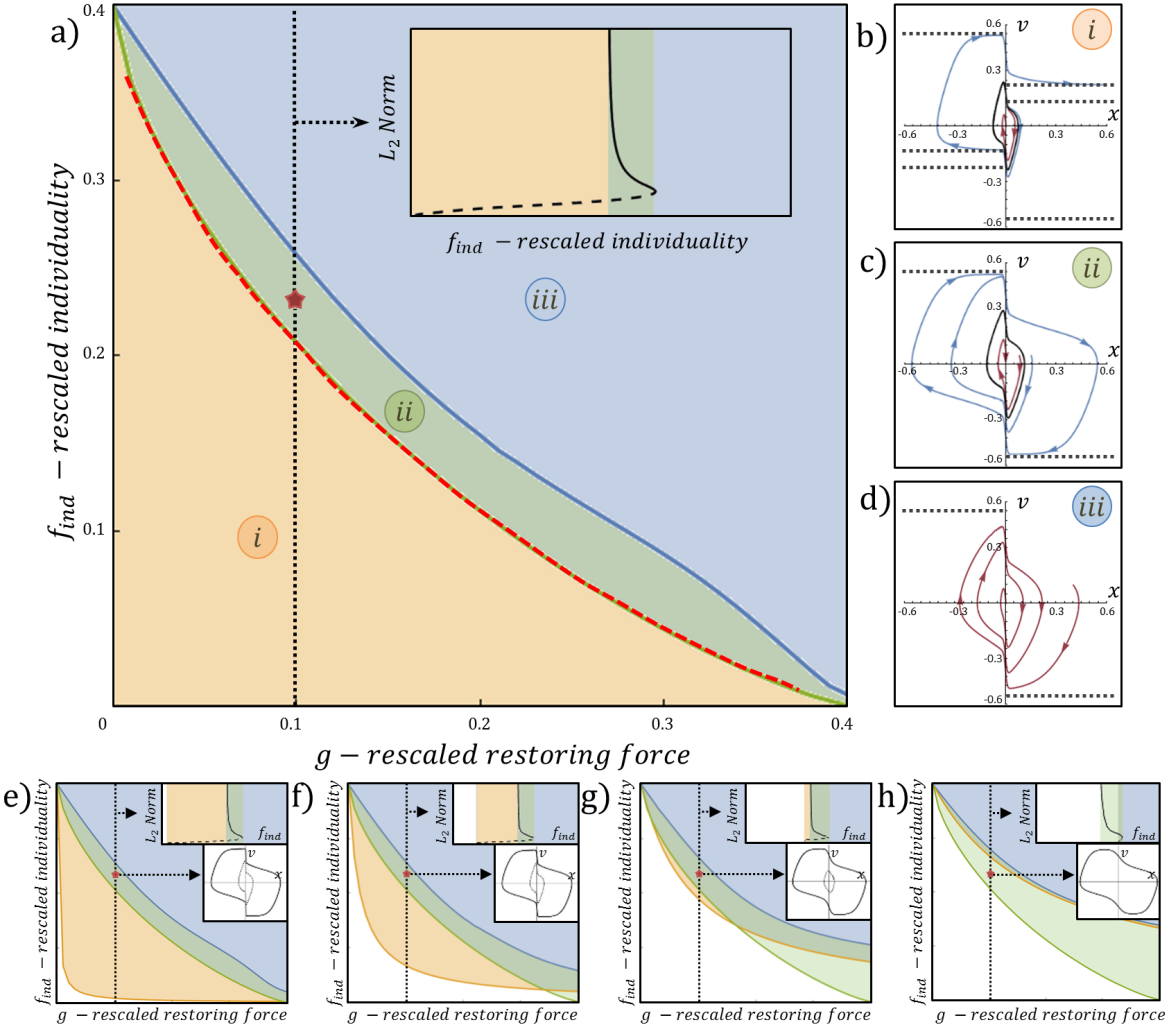
Stability analysis. (A) The *f*_*ind*_ − *g* phase diagram for *ϵ* → 0. Green line refers to the homoclinic bifurcation (Eq (12)). Blue line refers to the saddle node bifurcation. Top right inset displays the bifurcation diagram of *f*_*ind*_ along the dashed section where *g* = 0.1: *f*_*ind*_ against the (*x*, *v*) vector norm of the solution ($\sqrt{\int_{0}^{1}x{\left(t\right)}^{2}dt+\int_{0}^{1}x{\left(t\right)}^{2}dt}$), solid black line is the stable limit cycle and the dashed black line is the separatrix. Red marker refers to the point (*g*, *f*_*ind*_) = (0.1, 0.23). Red dashed line is the numerical estimation of the homoclinic bifurcation obtained using AUTO-07P [28]. (B-D) The {*x*, *v*} phase space portraits for phases (i), (ii), (iii), corresponding to the points (*g*, *f*_*ind*_) = (0.1, 0.18), (0.1, 0.23), (0.1, 0.28) respectively. Black dashed lines display the *q*(*x*, *v*) = 0 nullcline solutions. Black solid line display the separatrix. Red line display convergent flow. Blue line display free/oscillatory motion. (E-H) *f*_*ind*_ − *g* phase diagrams for *ϵ* = 0.001, 0.01, 0.05, 0.1 respectively. Orange line refers to the subcritical hopf bifurcation (Eq (10)). Green line refers to the homoclinic bifurcation (Eq (12)). Blue line refers to the saddle node bifurcation. Top right inset displays the bifurcation diagram of the dashed section line where *g* = 0.1. Middle right inset display the {*x*, *v*} phase space portrait that correspond to point (*g*, *f*_*ind*_) = (0.1, 0.23) represented by the red marker. In the middle insets, the solid/dashed black lines are the stable/unstable limit cycles. Other parameters used to produce the diagrams are in Table 1.

Stability analysis of (7–9) shows that the system has a single fixed point at (x*, v*) = (0, 0) (for full derivation see S1 Appendix). This fixed point undergoes a subcritical Hopf bifurcation for a critical value of *f*_*ind*_ given by

$$\begin{array}{c} f_{c}^{\left(1\right)}=\frac{n}{2}\left(\frac{1}{1+\frac{g}{2k_{c}\epsilon }}\right) \end{array}$$ (10)

where for $f_{ind}>f_{c}^{\left(1\right)}$ the fixed point is stable, and in the limit of *ϵ* → 0 (i.e, tanh(*x*/*ϵ*) → *sign*(*x*)), the fixed point is always stable ($f_{c}^{\left(1\right)}\to 0$). The *f*_*ind*_ − *g* phase diagram for the limit of *ϵ* → 0 (Fig 2A) displays three dynamical phases:

**(i) The low *f*_*ind*_ phase:** In this phase the system displays bi-stability between spiral convergent flow to the origin, and free motion to infinity at terminal velocity along the nullclines of *q*(*x*, *v*) (Fig 2B). These two modes of motion are separated by an unstable limit cycle, the seperatrix. As *f*_*ind*_ increases two of the *q*(*x*, *v*) nullclines approach each other in each half space of *x* (S1 Fig). At a critical value of $f_{ind}=f_{c}^{\left(2\right)}$ the system undergoes a homoclinic bifurcation, where the two nullclines merge and confine the free motion to a stable limit cycle. We find that the velocity at the transition is given by

$$\begin{array}{c} v^{\pm }=\pm g\mp \sqrt{\frac{n}{2}\left(\frac{n}{2}-f_{ind}\right)} \end{array}$$ (11)

where ± note the two halfspaces of *x*. By plugging (11) into the nullcline solution of (9) we obtain a transcendental form for the critical point of the homoclinic bifurcation (for full derivation see S1 Appendix)

$$\begin{array}{c} f_{c}^{\left(2\right)}=\frac{n}{2}sech^{2}\left(\frac{-g+\sqrt{\frac{n}{2}\left(\frac{n}{2}-f_{c}^{\left(2\right)}\right)}}{f_{c}^{\left(2\right)}}\right) \end{array}$$ (12)

**(ii) The intermediate *f*_*ind*_ phase:** For $f_{ind}>f_{c}^{\left(2\right)}$ we find bi-stability between relaxation oscillations [27] and convergent flow to the origin (Fig 2C). The velocity is bounded by the nullclines of (9) in each half space of *x*. As individuality *f*_*ind*_ increases, the limit cycle shrinks in size, and the seperatrix region expands (S2 Fig). At a critical value of $f_{ind}=f_{c}^{\left(3\right)}$ the system undergoes a saddle node bifurcation where the stable limit cycle coalesces with the separatrix and both collapse. The saddle node bifurcation critical line was evaluated using numerical continuation [28].

**(iii) The high *f*_*ind*_ phase:** For $f_{ind}>f_{c}^{\left(3\right)}$ the system spirals to the fixed point for every initial condition (no bi-stability). The phase space trajectories are shown in Fig 2D.

For finite and increasing values of *ϵ*, the sub-critical Hopf bifurcation line (Eq (10)) shifts to larger values of *f*_*ind*_ and *g* on the phase diagram, revealing larger regions where the fixed point is unstable with no bi-stability (Fig 2E–2H). Increasing *ϵ* also shifts the critical line of the saddle node bifurcation, yet the transition between phases (i) and (ii) remains unaffected. For large values of *ϵ*, where the pulling force of the informed ants, *G*, increases linearly when the cargo moves away from the opening, the system (7–9) displays a behavior similar to the tethered cargo [24], and is further discussed in S1 Appendix.

### The stochastic process schemes

The mean field description of our system (Eqs (7)–(9)) ignores the stochastic nature of the carrying ants. During the transport, the participating ants randomly attach/detach to/from the cargo, role switch between pulling and lifting, and switch from informed to uninformed ants [21]. Each of these processes introduces a new source of noise to the dynamics, and modifies the trajectories with respect to the deterministic solution. As a result, the applied force to the cargo fluctuates, which drives spontaneous transitions between the two modes of motion in the bi-stable regions of the phase diagram (Fig 2A).

**Fig 3:**
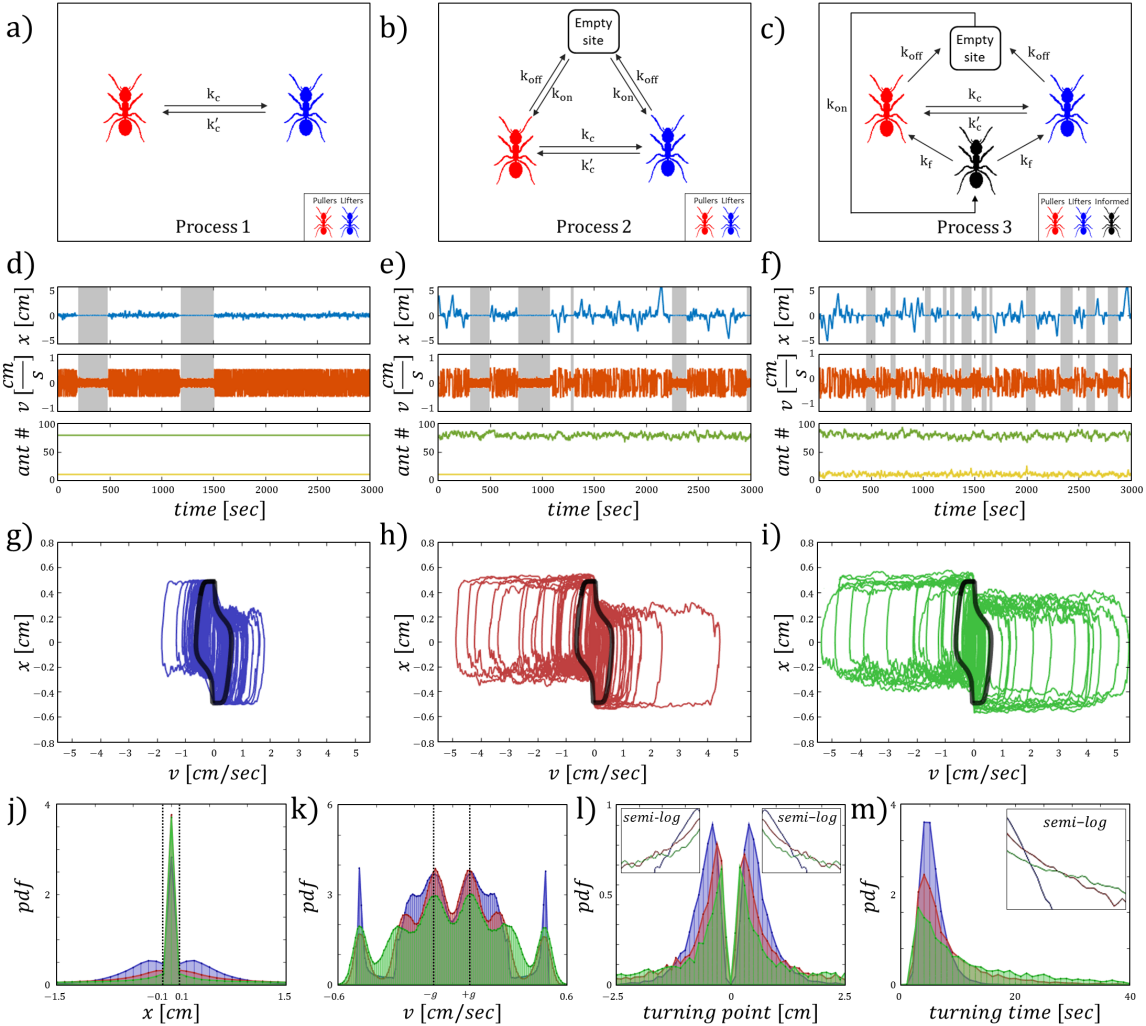
The stochastic process schemes. (A-C) Illustrations of the kinetics used in the simulations. (D-F) The dynamics of the three processes respectively. Upper panel is the time series of the position. Middle panel is the time series of the velocity. The gray sections cover the parts of the time series where the system is in a convergent mode of motion. Bottom panel shows the time series of the ant population on the cargo. Green/yellow represent the uninformed/informed ants respectively. (G-I) The {*x*, *v*} phase space trajectories for the three processes. Black solid line is the deterministic solution. (J) The position distributions, black dashed lines at ±0.1 are the thresholds for detecting convergence. (K) The velocity distributions, black dashed lines at ±*g* are the thresholds for detecting convergence. (L) The turning point distributions, upper panels display the semi-log plot of the probability density function for each half space of *x*. (M) The turning time distributions, upper right panel display the semi-log plot of the probability density function. Color index: Blue, red, green—processes 1, 2, 3 respectively.

To understand the effects of the different sources of force fluctuations (noise) on the dynamics in the bi-stable region (phase (ii), Fig 2C), we simulated the motion of the cargo using a Gillespie algorithm [29], for three different kinetic schemes (see Methods for algorithm):

**(i) Role switching:** In this scheme, the cargo binding sites are fully occupied by *n*_*tot*_ uninformed ants at all time. Therefore, the motion of the cargo results from role switchings between pullers and lifters with respect to Eqs (1) and (2). The number of informed ants *G* is constant, and they act as a restoring force pulling towards the origin (Fig 3A).

**(ii) Attachments/detachments:** This scheme allows attachments/detachments of uninformed ants to/from the cargo binding sites with constant rates *k*_*on*_/*k*_*off*_ respectively, such that the number of uninformed ants that role switch with respect to Eqs (1) and (2) fluctuate around *n*_*tot*_. The number of informed ants, *G*, is constant, and they act as a restoring force towards the origin (Fig 3B).

**(iii) Informed pullers:** In the final scheme, newly attached ants act as informed pullers that apply their force towards the origin. The informed ants convert to uninformed ants at a constant rate *k*_*forget*_ [21]. The number of informed ants fluctuate around a mean value *G*, and the number of uninformed ants, that can attach/detach and role switch, fluctuate around a mean value of *n*_*tot*_ (Fig 3C).

All three processes initiate spontaneous transitions between the two modes of motion (Fig 3D–3F). Furthermore, as the number of noise sources increase, the oscillatory motion becomes irregular, with larger amplitude excursions that deviate from the stable limit cycle (Fig 3G–3I). During these excursions the mean velocity is almost unchanged, and is approximately on the nullcline values of the deterministic model (Fig 2C). The deviations from the predicted stable limit cycle can be attributed to the fluctuations in *n*_*tot*_ and *G*. When the number of ants fluctuate, the force applied to the cargo fluctuate as well: for example, a transient increase in the number of uninformed ants and/or a transient decrease in the number of informed ants leads to an increase in group persistence. When the motion is more persistent, this leads to larger amplitude excursions away from the opening (Fig 3G–3I).

Analysis of the motion, made over many realizations, reveals several features of the bi-stable behavior: (1) The distributions of the position (Fig 3J) display a large peak centered around *x* = 0, which corresponds to the convergent mode of motion. (2) The distributions of the velocity (Fig 3K) show peaks that correspond to the oscillatory motion along the nullclines, and to ±*g* when the motion is convergent in the proximity of *x* = 0. (3) The distributions of the turning point and turning time (Fig 3L and 3M) are found to have an exponential tail, indicating that random, statistically independent fluctuations, limit the motion when it extends beyond the predicted stable limit cycle amplitude. These distributions display longer tails as the number of stochastic processes increase and correspond to larger amplitude excursions. These results provide a mechanistic explanation for the exponential relation between the first arrival time at each position along the obstacle and the distance of that position from the opening [30], that was observed in previous experiments [22].

The convergent mode of motion was identified by applying the following thresholds over the time series: (i) Position: −0.1 < *x* < 0.1 [cm], as indicated by the sharp peak near the origin (Fig 3J). (ii) Velocity: −0.1 < *v* < 0.1 [cm/s], which is the magnitude of ±*g* (Fig 3K). (iii) Duration: *t* > 5 [sec], a sufficient period time to distinguish between a convergent flow and oscillations that pass near the origin.

### Physical conditions for dynamical bi-stability

The physical process that prompts the bi-stability in phases (i) and (ii) is the following: Consider a system initially at rest (*v*|_*t* = 0_ = 0) at a location near the origin that is bound by the seperatrix (Fig 2B and 2C). The system will be pulled by a force with magnitude *g* towards the origin and begin to order the internal pulling forces of the uninformed ants by switching their roles (Eqs (1) and (2)). However, when passing the origin, the external force changes its direction abruptly, which disrupts the ordering process. If by the time the group passes the origin the pulling forces of the uninformed ants are insufficient for crossing the separatrix, the abrupt change in direction of *g* will cause the group to lose its order by switching roles in the opposite direction, slow down and spiral to the origin. For a sufficiently large initial distance outside the seperatrix, the system is able to order the pulling forces sufficiently, and overcome the restoring force to continue in a ballistic motion (phase (i)) or perform relaxation oscillations (phase (ii)).

**Fig 4:**
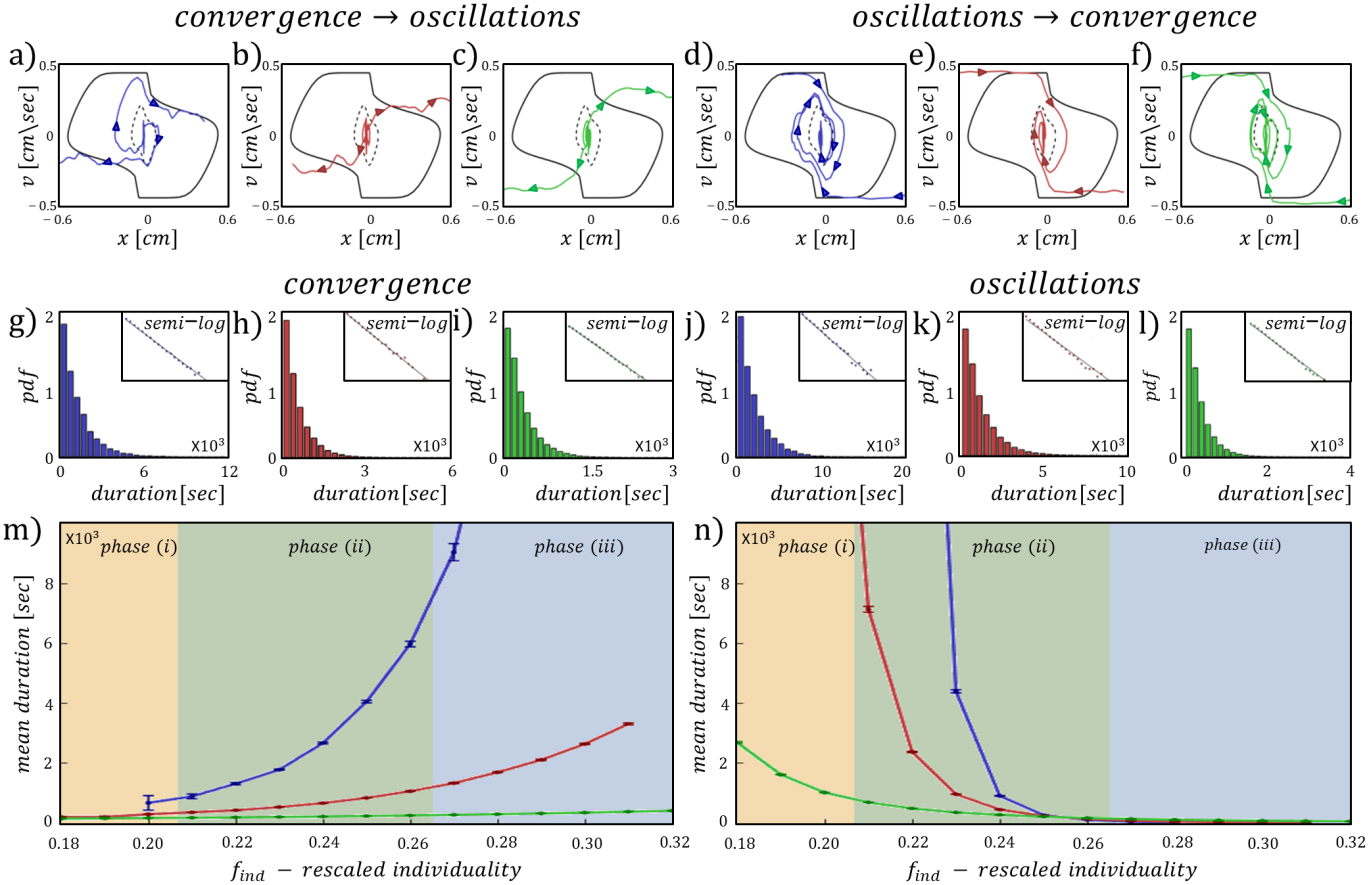
The transitions between the bi-stable modes. Color index: Blue, red, green—processes 1, 2, 3 respectively. (A-C) {*x*, *v*} phase space trajectories at the transition between convergence to oscillations. (D-F) {*x*, *v*} phase space trajectories at the transition between oscillations to convergence. Black solid/dashed line is the deterministic stable/unstable limit cycle. Plots correspond to the point (*f*_*ind*_, *g*) = (0.23, 0.1) represented by the red marker on the *f*_*ind*_ − *g* phase diagram (Fig 2A). (I-K) The distributions of durations spent in the convergent mode of motion. (J-L) The distributions of durations spent in the oscillatory mode of motion. Upper right insets display the semi-log plots of the probability density functions. The distributions correspond to the point (*g*, *f*_*ind*_) = (0.1, 0.23) represented by the red marker on the *f*_*ind*_ − *g* phase diagram (Fig 2A). (M-N) The mean duration spent in convergent/oscillatory motion with respect to the rescaled individuality *f*_*ind*_ in the proximity of region (ii) on the phase diagram (Fig 2A). Fill colors (orange, green, blue) represent the three phases.

The phase space trajectories from the simulations show that the transitions between the modes of motion occur near the origin, due to the fluctuations in the force applied to the cargo (Fig 4A–4E):

**• Convergence → oscillations** (Fig 4A–4C): These transitions occur when the system accelerates and reaches its maximal velocity near the origin. When passing the origin, the restoring force changes sign and the system starts to slow down. However, at this point large enough fluctuations can kick the system out of the separatrix convergent domain, before it has slowed down significantly due to *g*.

**• Oscillations → Convergence** (Fig 4D–4E): The system approaches the opening at *x* = 0 with maximal velocities close to the nullcline solutions of (9) in either half spaces of *x*. When the system passes the origin it experiences a drop in velocity by ∼2*g*. This abrupt change in velocity, with an addition of a fluctuation, can drop the velocity low enough to transit the system into the stable region of convergence.

The distributions of the duration spent in each mode of motion has an exponential form (Fig 4G–4L), showing that the transitions between the two modes of motion are stochastic, driven by fluctuations of the applied force to the cargo. We show that within phase (ii), as *f*_*ind*_ increases, the mean duration spent in convergence increases (Fig 4M), while the mean duration spent in oscillations decreases (Fig 4N). These results demonstrate that an increase in *f*_*ind*_ increases the probability to transit from oscillations to convergence when the motion is persistent. Since an effective increase in *f*_*ind*_ is achieved experimentally by decreasing the system size [21], these results indicate that smaller carrying groups have a higher probability to display co-existence between the two modes of motion in the presence of an obstacle.

### Experiments and 2D simulations

To test our predictions of dynamical bi-stability during cooperative transport in the presence of an obstacle, we have conducted experiments using ring-like cargoes of different sizes [21]. These cargoes were similarly treated with cat food, which made them equally attractive (see Methods). In addition, the number of binding sites were equally distributed along the perimeter of the cargo rings, which made the number of carrying ants *n*_*tot*_ proportional to the radius of the cargo *r*, i.e, *n*_*tot*_ ∝ *r*.

The motivation for using different cargo sizes arises from its effect on the level of cooperativity between the carrying ants [21, 24], which in our model is set by the individuality parameter *F*_*ind*_, and the total applied force *f*_*tot*_ (Eqs (1) and (2)). While *F*_*ind*_ is independent of object size, the total applied force *f*_*tot*_ is proportional to the number of carrying ants *n*_*tot*_, which make *f*_*tot*_ ∝ *r*. Moreover, the mechanical response coefficient *γ* ∝ *r* as well (see S1 Appendix for scaling arguments). Consequently, the dynamics of the cargo (Eq (9)) depend on the rescaled parameter, *f*_*ind*_, as *f*_*ind*_ = *F*_*ind*_/*γ* ∝ 1/*r*.

Therefore, changing the cargo size allows us to effectively probe different levels of rescaled individuality *f*_*ind*_, and use cargo size as a means for manipulating the cooperativity level between the ants. In addition, changing the cargo size effectively controls the parameter *ϵ* in our model (Fig 1C), as a decrease in *r* effectively decreases *ϵ*, and exposes a larger region of bi-stability in the predicted phase diagram (Fig 2E–2H).

**Fig 5:**
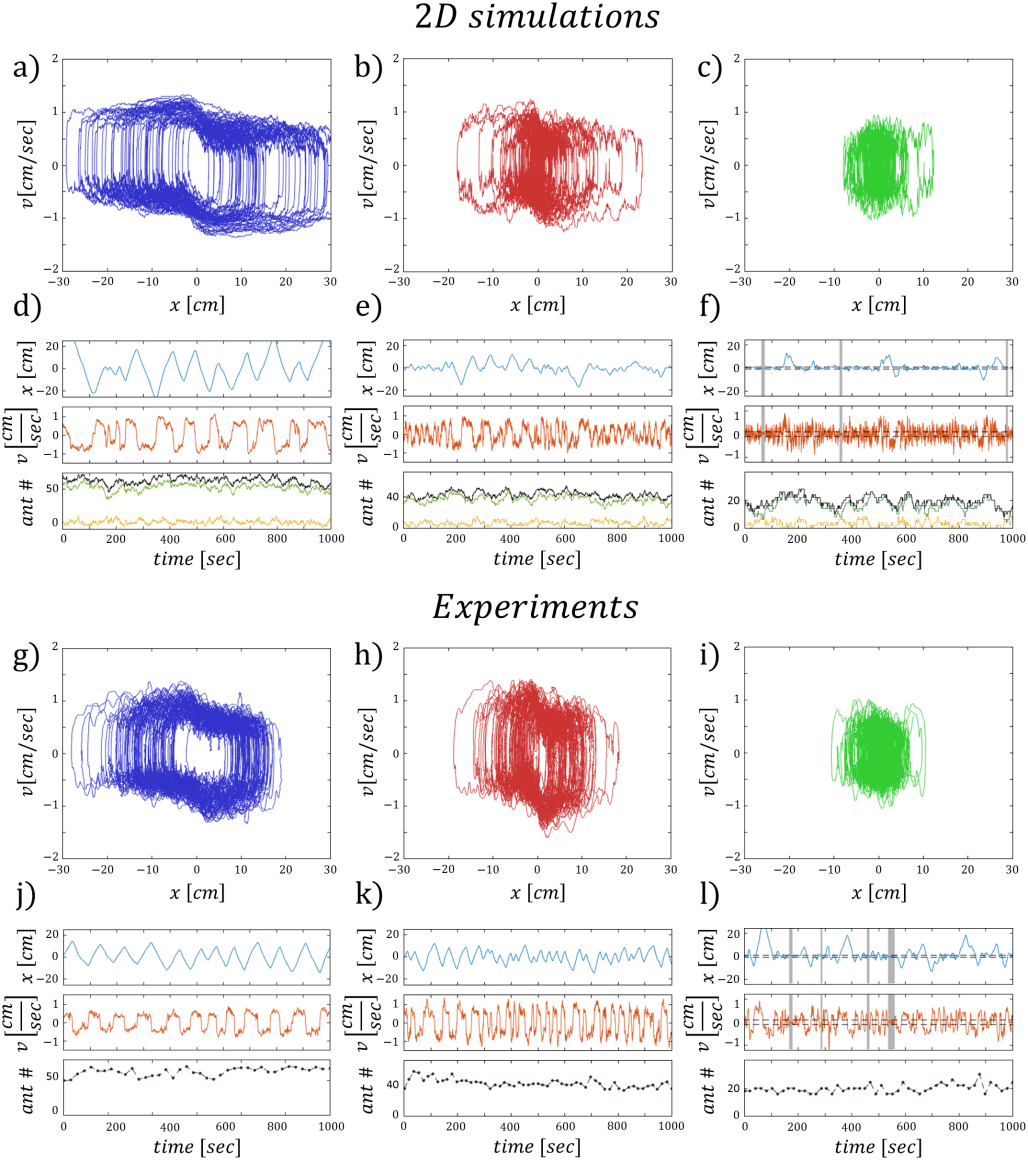
Experiments and two dimensional simulations. 2D Simulations: (A-C) {*x*, *v*} phase space trajectories for the motion of the three cargoes. (D-F) Cargo dynamics. Upper panel displays the position time series. Middle panel displays the velocity time series, gray sections cover the convergent mode of motion. Black dashed lines indicate the thresholds for detecting convergence. Bottom panel displays the time series of the ant population on the cargo. Green/yellow represent the uninformed/informed ants respectively. Black is the total number of ants carrying the cargo. Experiments: (G-I) {*x*, *v*} phase space trajectories for the motion of the three cargoes. (J-L) Cargo dynamics. Upper panel displays the position time series. Middle panel displays the velocity time series, gray sections cover the convergent mode of motion. Black dashed lines indicate the thresholds for detecting convergence. Bottom panel display the time series of the ant population on the cargo. Parameters for the simulations are given in Table 1. Color index: Blue, red, green—large, medium, small cargoes (*R* = 2, 1, 0.5 [cm]) respectively.

We recorded ants carrying cargoes of three sizes in the experimental setup shown in Fig 1A (see Methods for full set-up). The experiments were compared to two dimensional simulations that reproduced the same experimental features (see S1 Appendix for 2D mathematical model). The results show similar trends as function of cargo size in both simulations and experiments (Fig 5):

**• Large cargo** (*r* = 2 [cm]): For this cargo size the motion is the most persistent, and the phase space trajectories show clear non-linear relaxation oscillations. The step-like changes in the velocity are observed to occur when passing the opening (Fig 5A–5D).

**• Medium cargo** (*r* = 1 [cm]): Here, the phase space trajectories demonstrate non linear relaxation oscillations as well, however with a smaller amplitude compared to the large cargo (Fig 5E–5H).

**• Small cargo** (*r* = 0.5 [cm]): This size is approximately the size of common natural prey [21]. For this cargo the phase space trajectories demonstrate noisy oscillations, as well as dense trajectories near the origin. These results suggest that the system spends a significant time near the opening (Fig 5A–5C and 5G–5I).

**Fig 6:**
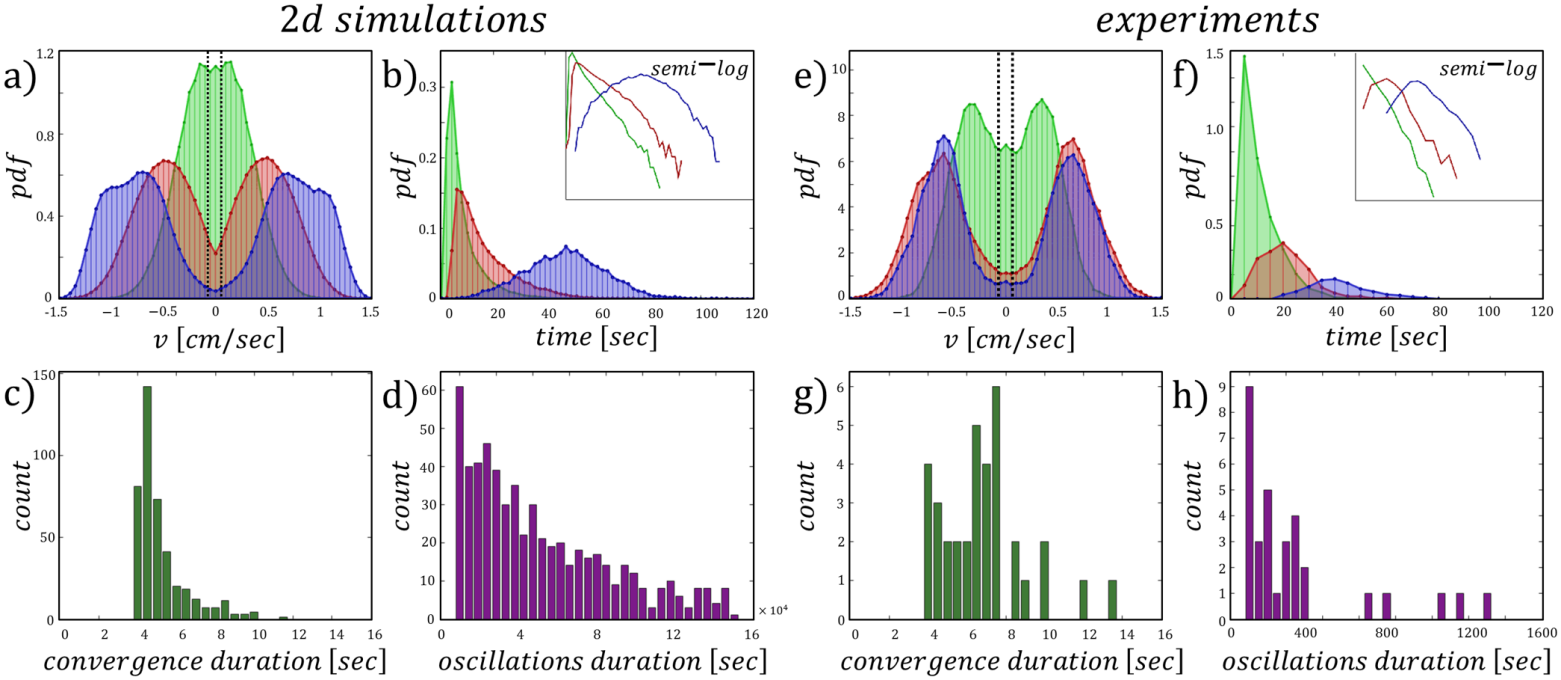
Evidence of bi-stability in experiments and two dimensional simulations. (A) Velocity distributions. Black dashed lines indicate the threshold for detecting convergence. (B) Turning time distributions. Upper right insets display the semi-log plots. (C-D) Distribution of the duration spent by the small cargo in the convergent/oscillatory mode with mean, variance of 6.8, 4.6 [sec]/5.6 × 10^4^, 3.6 × 10^9^ [sec] respectively. Data analysis of experiments: (E) Velocity distributions. Black dashed lines indicate the threshold for detecting convergence. (F) Turning time distributions. Upper right insets display the semi-log plots. (G-H) Distribution of the duration spent by the small cargo in the convergent/oscillatory mode of motion with mean, variance of 5.1, 1.6 [sec]/250, 1.2 × 10^6^ [sec] respectively. Parameters for the simulations are given in Table 1. Color index: Blue, red, green—Large, medium, small cargoes.

The turning time distributions of the medium and small cargoes have an exponential tail (Fig 6B and 6F), similar to the one dimensional simulations (Fig 3M), and in agreement with the first passage time observed in [22]. The large cargo however, has a rather well defined period, indicating that for a large system size the oscillatory mode of motion dominates.

For the smallest cargo, we find that the velocity distribution peaks at *v*_*x*_ = 0 in both simulations and experiments (Fig 6A and 6E). This peak appears in addition to peaks that represent the oscillatory motion, and are observed for all cargo sizes. This result suggests that for small cargoes, the system exhibits two co-existing modes of motion with different characteristic velocities, similar to the 1D simulations (Fig 3K).

To detect the time the small cargo spends in the convergent mode near the opening (Fig 6C–6L), we applied the following thresholds over both the experimental and simulation data: (i) Position threshold: Over the *x* and *y* time series, the thresholds were set to −*r* < *x*_*conv*_ < *r* and *y*_*conv*_ < 2*r*. These thresholds consider the cargo size. (ii) Velocity threshold: The threshold over the velocity *v*_*x*_ time series considers the theoretical velocity given by the pull of a single ant in the absence of friction, and in a direction parallel to the obstacle. In such a case *v*_*x*_ = *f*_0_/*γ* ≈ 0.14 [cm/sec] (Table 1). Considering that fluctuations are dominant for this system size, the threshold was set to *v_x_conv__* < 0.1 (Fig 6A and 6C). (iii) Duration threshold: Convergent events were counted only if surpassed the *x*, *y*, *v*_*x*_ thresholds and lasted more than 4 seconds. The threshold *t*_*conv*_ > 4 [sec] assured a sufficient period of time dwelling near the opening, that can be distinguished from an oscillatory motion at low velocity.

**Table 1:** Default parameters used in the 1D and 2D simulations and the analytical model.

	1D model and simulations				2D simulations		
Parameter	1D model	Process 1	Process 2	Process 3	*r* = 2 [cm]	*r* = 1 [cm]	*r* = 0.5 [cm]
*n*_*max*_	80	80	100	110	96	48	16
*n*_*tot*_	80	80	100	110	52.5	25.8	8.6
*G*	10	10	10	10	8.5	4.2	1.4
*k*_*c*_	1	1	1	1	1	1	1
*k*_*on*_	-	-	0.06	0.06	0.0214	0.0214	0.0214
*k*_*off*_	-	-	0.015	0.015	0.015	0.015	0.015
*k*_*forget*_	-	-	-	0.12	0.09	0.09	0.09
*k*_*orient*_	-	-	-	-	0.7	0.7	0.7
*f*_0_	1	1	1	1	2.8	2.8	2.8
*F*_*ind*_	23	23	23	23	28	28	28
*γ*	100	100	100	100	25 ⋅ *scale*	25 ⋅ *scale*	25 ⋅ *scale*

Counting the durations spent in each mode of motion suggests an exponential trend in the simulations (Fig 6C and 6D). The limited experimental data did not allow us to extract the type of distribution (Fig 6G and 6H), however, the trend seems exponential, and both experiments and simulations provide similar durations of convergent events. These results show, as predicted by our model, that a small group may exhibit co-existing dynamical bi-stability, and that a decrease in the cargo size leads to a decrease in group persistence, along with an increase in the duration of convergent motion.

In S1 Video we show a typical dynamical bi-stability in the two dimensional simulations, with parameters that correspond to phase (ii) (Fig 2). In S2 and S3 Videos we show the typical oscillatory motion of the large and medium size cargoes (corresponding to the data shown in Fig 5G and 5H). Finally, in S4 Video we provide examples of transitions between oscillations and convergent motion when the ants carry the small cargo (corresponding to the data shown in Fig 5I).

## Discussion

**Fig 7:**
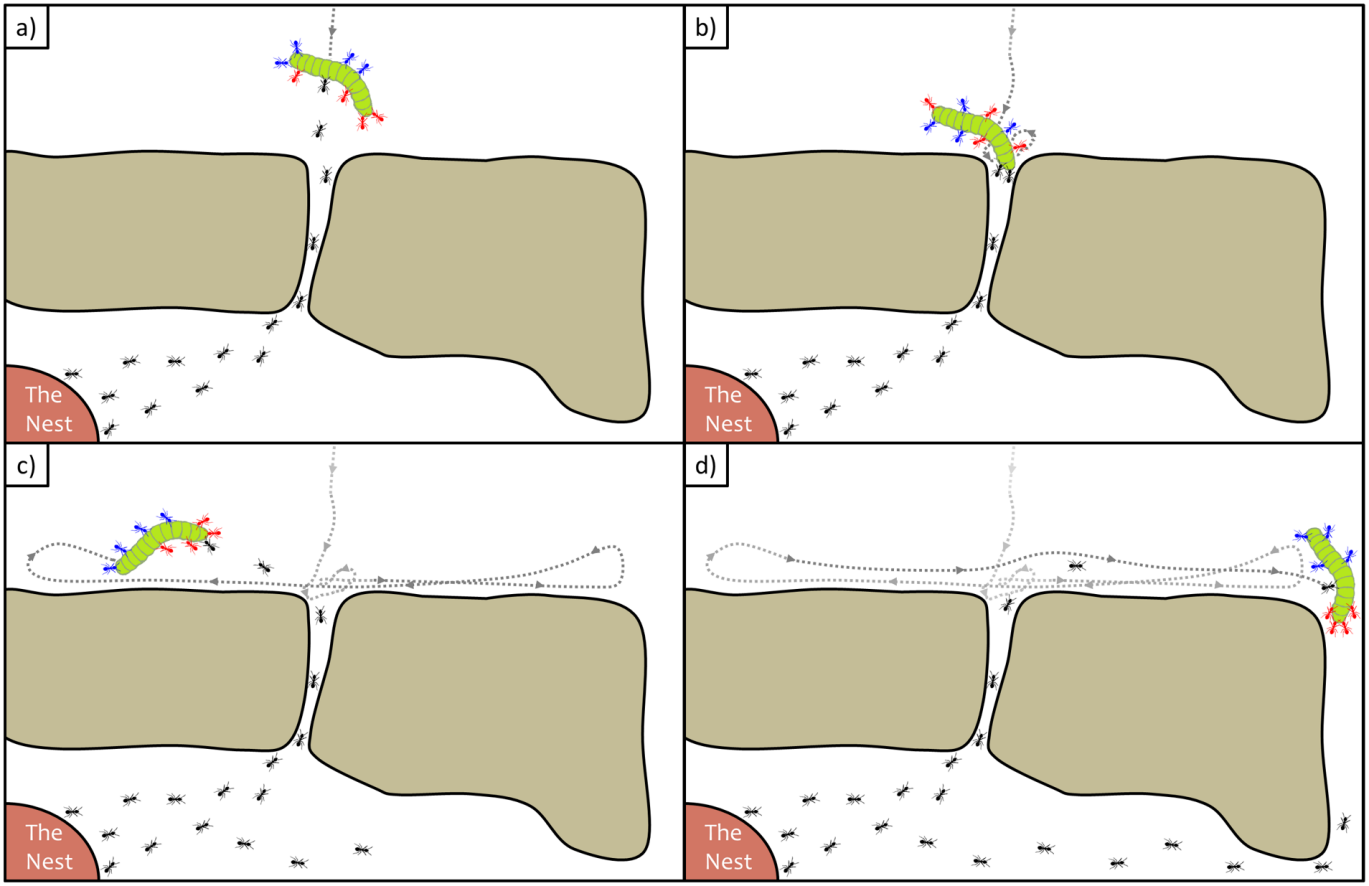
The conflict that ants face when encountering an obstacle during food retrieval to the nest. (a) Informed ants direct the carrying group towards the pheromone scent trail that pass through the opening and as a result encounter the obstacle. (b) The ants dwell near the opening and the motion is dominated by informed ants that attempt to squeeze the cargo through. (c) The ants motion is dominated by the uninformed ants that attempt to align their direction of motion and perform sideways oscillations. (d) The ants perform a large amplitude excursion that take them across the barrier to meet other informed individuals that mark a new pheromone scent trail.

During cooperative transport of food, ants often encounter obstacles, such as barriers with small openings that allow single ants to pass, but are too narrow for the food to be retrieved [21, 22]. These encounters raise a conflict within the carrying group: While the group attempt to maintain a persistent motion that will allow them to bypass the obstacle, informed individuals attempt to direct the load towards the opening, which is identified as the direct route to the nest. In this work, we explain the behavior of ant groups near obstacles, using a physical mechanism that originates from the mechanical interactions between the individuals in the group. We show that when a group of ants encounters such an obstacle during cooperative transport (Fig 7A), two modes of motion spontaneously emerge. These two modes of motion can assist the group in overcoming the obstacle: Either by dwelling near an opening, which may allow flexible loads to be squeezed through the direct route to the nest (Fig 7B), or, when carrying large items of food, perform persistent excursions (Fig 7C), which may lead to obstacle circumvention. (Fig 7D). With the use of experiments and simulations, we demonstrate how intrinsic noise, in the form of mechanical fluctuations, allows stochastic transitions between the two modes of motion, and keeps the system out of detrimental behaviors, such as remaining stuck near an opening with large loads that cannot be squeezed through, or oscillating for long durations, when carrying loads that can be passed through the opening.

Previous studies of cooperative transport by ants in the presence of constraints have investigated the behavior of the carrying ant group near obstacles with open boundaries [21, 22, 25], a fully confining obstacle that trap the group [25], and by confining the motion of the cargo by a tether [24]. These studies have related the decision making process of the individual ants to problem solving behaviors during obstacle navigation [21, 22, 24], and proposed a strategy in which the ants’ behavior changes over the course of time when facing obstacles [25]. Here, we further examine the interaction of ant groups with obstacles, using a simple experimental setup that allows a detailed analysis of the cargo’s motion. Our results provide direct evidence for the emergence of dynamical bi-stability in the presence of rigid obstacles, and elaborates further the current physical understanding of cooperative transport by ants [21, 22, 24]. In addition, the analytical framework displayed in this work provides a detailed explanation of the origin and conditions for maintaining co-existence between the two collective dynamical modes. These findings could have implications for other biological ensembles [2–5] and theoretical models [31, 32], that have reported co-existence between several collective dynamical modes.

## Methods

### Experimental setup

**Fig 8:**
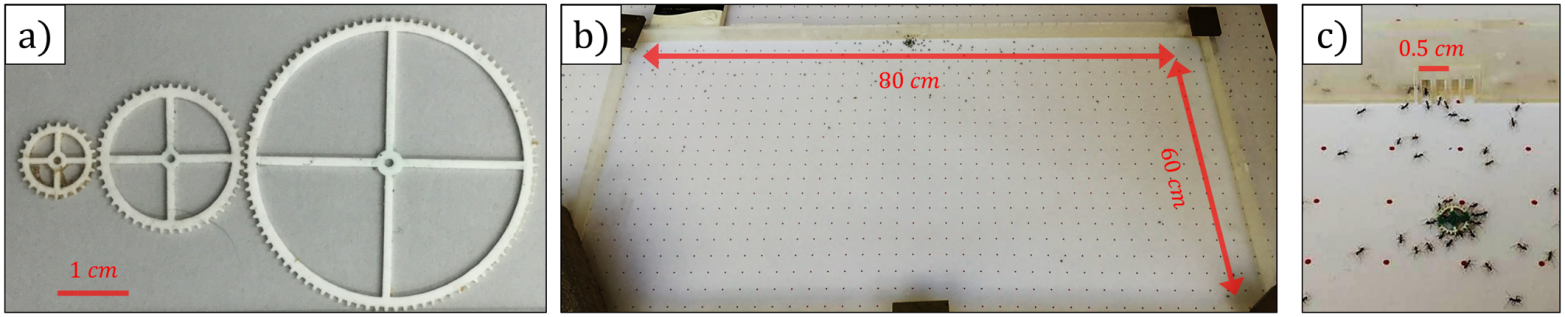
The experimental setup. The experimental setup. (a) The cargoes. (b) The closed frame. (c) The opening used for the small cargo experiments (*r* = 0.5), two of the four slits were sealed.

Experiments were carried out in a single colony of *P. longicornis* ants in Rehovot, Israel during the summer months (June—September) at day time (between 9:00-14:00) when the foraging activity is high [13]. Conducting these experiments on a single large colony was sufficient, as it was previously found that cooperative transport behavior in these ants is similar among different colonies [21, 22, 24]. Cooperative transport was tested with three ring shaped cargoes with radii *r* = 0.5, 1, 2 [cm] and width 0.1 [mm] (Fig 8A). In each experiment the ants were filmed from a top vertical view, using a Sony FDR-AX100 4K Ultra HD Camcorder camera. An enclosed 80 × 60 [cm] PMMA frame with a opening was placed on the flat surface with a graph paper on top, such that the opening faced the direction of the nest (Fig 8B). The frame was sufficiently large to mimic an infinite length obstacle, as the carrying groups never reached the frame edges with all cargo sizes. Each experiment started with a recruitment phase, where ants were lured from the nest with several fresh pieces of cat food placed at the proximity of the opening. Once a recognizable trail could be identified, and a significant amount of ants were seen milling through the camera, the food was replaced by a circular cargo smeared with cat food. Each experiment lasted approximately an hour. A total of 15 experiments were conducted, 5 experiments for each cargo size. The opening has a width of 0.5 [cm]. For the experiments of the small cargo where *r* = 0.5 [cm], the hole was shaped as a comb such that the cargo cannot get physically stuck at the opening (Fig 8C). Tracking was carried over the cargo center of mass using a designated software developed on Matlab.

### Parameter estimation

Two of the free parameters were obtained directly from the experiments: The detachment rate *k*_*off*_ [21], and the average number of carrying ants occupying the cargo. The rest of the parameters were estimated by considering the steady state populations of the informed and uninformed ants. For the second stochastic scheme (Fig 3C), the steady state population of the uninformed ants *P*_*u*_ is given by

$$\begin{array}{c} \frac{dP_{u}}{dt}=k_{on}\left(1-P_{u}\right)-k_{off}P_{u}⟶P_{u}=\frac{k_{on}}{k_{on}+k_{off}} \end{array}$$ (13)

Therefore the total number of binding sites can be estimated by:

$$\begin{array}{c} P_{u}=\frac{n_{tot}}{n_{max}}⟶n_{max}=n_{tot}\left(1+\frac{k_{off}}{k_{on}}\right) \end{array}$$ (14)

For the third stochastic scheme (Fig 3C), and the two dimensional simulations, the steady state populations of the informed/uninformed ants, *P*_*inf*_/*P*_*u*_ are given by

$$\begin{array}{c} \frac{dP_{inf}}{dt}=k_{on}\left(1-P_{inf}-P_{u}\right)-k_{forget}P_{inf}⟶P_{inf}=\frac{1}{1+\kappa_{1}+\kappa_{2}} \end{array}$$ (15)

$$\begin{array}{c} \frac{dP_{u}}{dt}=k_{forget}P_{inf}-k_{off}P_{u}⟶P_{u}=\frac{\kappa_{1}}{1+\kappa_{1}+\kappa_{2}} \end{array}$$ (16)

where *k*_*forget*_ is the rate at which informed ants convert to uninformed, $\kappa_{1}=\frac{k_{forget}}{k_{off}}$ and $\kappa_{2}=\frac{k_{forget}}{k_{on}}$. Therefore the number of binding sites can be estimated by:

$$\begin{array}{c} \frac{n_{tot}+G}{n_{max}}=P_{u}+P_{inf}⟶n_{max}=\left(n_{tot}+G\right)\left(1+\frac{\kappa_{2}}{1+\kappa_{1}}\right) \end{array}$$ (17)

and the forget rate was estimated by

$$\begin{array}{c} k_{forget}=\frac{\left(\frac{n_{max}}{G+\langle n_{tot}\rangle }-1\right)k_{on}}{1-\frac{k_{on}}{k_{off}}\left(\frac{n_{max}}{G+\langle n_{tot}\rangle }-1\right)} \end{array}$$ (18)

The full parameter list is given in Table 1.

### Gillespie algorithm

The simulation of the dynamics is based on a Gillespie algorithm [29], where in each iteration of the simulation, one of the following events can occur:

- Attachment of an informed ant to an empty site.
- Detachment of an uninformed puller/lifter from an occupied site.
- Role switching between the uninformed pullers/lifters.
- Conversion between an informed puller to an uninformed puller/lifter.
- Re-orientation of an un-informed puller ant towards the direction of motion of the cargo.

**The first step:** The time step of the next event is calculated by:

$$\begin{array}{c} dt=\frac{1}{R_{tot}}log\left(\frac{1}{r_{1}}\right) \end{array}$$ (19)

where *r*_1_ is drawn from a uniform distribution and *R*_*tot*_ is the sum of all possible rates given by

$$\begin{array}{c} R_{tot}=R_{att}+R_{det}+R_{dec}+R_{forget}+R_{orient} \end{array}$$ (20)

The rates in Eq (20) are given by

$$\begin{array}{c} R_{att}=k_{on}\cdot n_{empty} \end{array}$$ (21)

$$\begin{array}{c} R_{det}=k_{off}\cdot \left(n_{p}+n_{l}\right) \end{array}$$ (22)

$$\begin{array}{c} R_{dec}=k_{c}\sum_{i=0}^{n_{max}}\left|\alpha_{i}^{k}\right|exp\left(\alpha_{i}^{k}\frac{{\vec{f}}_{tot}\cdot {\vec{p}}_{i}}{F_{ind}}\right) \end{array}$$ (23)

$$\begin{array}{c} R_{forget}=k_{forget}\cdot G \end{array}$$ (24)

$$\begin{array}{c} R_{orient}=k_{orient}\cdot n_{p} \end{array}$$ (25)

where *k*_*on*_, *k*_*off*_, *k*_*c*_, *k*_*forget*_, *k*_*orient*_ are the constant basal rate constants of attachment, detachment, role switching, forgetting and reorientation respectively. *G*, *n*_*p*_, *n*_*l*_, *n*_*empty*_ are the average numbers of informed ants, pullers, lifters and empty sites respectively.

**The second step:** The type of event at time *t*+*dt* is determined by the following conditional statement:

If $\left(r_{2}<\frac{R_{att}}{R_{tot}}\right)$ then an informed ant attaches to the cargo

else if $\left(\frac{R_{att}}{R_{tot}}<r_{2}<\frac{R_{att}+R_{det}}{R_{tot}}\right)$ then an uninformed ant detaches from the cargo

else if $\left(\frac{R_{att}+R_{det}}{R_{tot}}<r_{2}<\frac{R_{att}+R_{det}+R_{dec}}{R_{tot}}\right)$ then an uninformed ant role switch

else if $\left(\frac{R_{att}+R_{det}+R_{dec}}{R_{tot}}<r_{2}<\frac{R_{att}+R_{det}+R_{dec}+R_{forget}}{R_{tot}}\right)$ then an informed ant turns into an uninformed ant

else an uninformed ant orients its position with respect to the total force vector. The numbers *r*_2_ is drawn at each step from a uniform distribution.

**The third and final step:** The position and velocity are updated by

$$\begin{array}{c} {\vec{v}}_{t+dt}={\vec{v}}_{t}+\frac{{\vec{f}}_{tot}}{\gamma } \end{array}$$ (26)

$$\begin{array}{c} {\vec{x}}_{t+dt}={\vec{x}}_{t}+{\vec{v}}_{t+dt}dt \end{array}$$ (27)

Note that in the cases where an informed ants “forgets” or an uninformed ant detaches, the type of uniformed ant (puller or lifter) should be determined as well. For such cases, another number *r*_3_ is drawn from a uniform distribution and the following conditional statement takes place:

**if** $\left(r_{3}<\frac{1}{1+exp(-2\frac{{\vec{f}}_{tot}\cdot {\vec{p}}_{i}}{F_{ind}})}\right)$ **then** an un-informed puller will participate

**else** an un-informed lifter will participate

The last conditional statement, results from the assumption of role switching equilibrium (at each site *i*)

$$\begin{array}{c} n_{p}^{i}exp\left(\frac{{\vec{f}}_{tot}\cdot {\vec{p}}_{i}}{F_{ind}}\right)=n_{l}^{i}exp\left(-\frac{{\vec{f}}_{tot}\cdot {\vec{p}}_{i}}{F_{ind}}\right) \end{array}$$ (28)

and by denoting the average number of pullers and lifters $N_{p}=\frac{n_{p}^{i}}{n_{p}+n_{l}}$ and $N_{l}=\frac{n_{l}^{i}}{n_{p}+n_{l}}$, we get

$$\begin{array}{c} N_{p}exp\left(\frac{{\vec{f}}_{tot}\cdot {\vec{p}}_{i}}{F_{ind}}\right)=\left(1-N_{p}\right)exp\left(-\frac{{\vec{f}}_{tot}\cdot {\vec{p}}_{i}}{F_{ind}}\right) \end{array}$$ (29)

which results in the required condition

$$\begin{array}{c} N_{p}=\frac{1}{1+exp(-2\frac{{\vec{f}}_{tot}\cdot {\vec{p}}_{i}}{F_{ind}})} \end{array}$$ (30)

Therefore, if *r*_3_ < *N*_*p*_, the ant will switch to a puller, and if *r*_3_ > *N*_*p*_, the ant will switch to a lifter.

## Supporting information

S1 Appendix Full derivation and analysis of the analytical model. (PDF)

S1 Fig The homoclinic bifurcation. Before the transition (*f*_*ind*_ = 0.18): (a) Position and velocity as a function of time. (b) Phase space trajectories (blue) and nullclines (dashed black). (c) The solution of *q*(*v*, *x*) for each half space of *x* under the approximation of *ϵ* → 0. After the transition (*f*_*ind*_ = 0.21): (d) Position and velocity as a function of time. (e) Phase space trajectory (blue) and nullclines (dashed black). (f) The solution of *q*(*v*, *x*) for each half space of *x* under the approximation of *ϵ* → 0.
(TIF)

S2 Fig The saddle node bifurcation. Solutions of the deterministic model along the oscillatory bi-stable region for parameters *g* = 0.1 and *f*_*ind*_ = 0.22, 0.23, 0.24, 0.25. (a-d) Position and velocity time series, blue curve is the position, red curve is the velocity. (e-h) Phase space trajectories, solid line is the stable limit cycle, dashed line is the unstable limit cycle (separatrix).
(TIF)

S1 Video Two dimensional simulation—bistable dynamics. (MP4)

S2 Video Large cargo experiment—persistent oscillations. (MP4)

S3 Video Medium cargo experiment—persistent oscillations. (MP4)

S4 Video Small cargo experiment—dynamical bi-stability. (MP4)
