# Supplementary material for: Bi-stability in cooperative transport by ants in the presence of obstacles
Source: PLoS Comput Biol. 2018 May 10;14(5):e1006068. doi: 10.1371/journal.pcbi.1006068 (PMC5944914; doi:10.1371/journal.pcbi.1006068)
Supplement: S1 Appendix — (PDF) [file pcbi.1006068.s001.pdf]

Supporting information appendix

Jonathan E. Ron[1], Itai Pinkoviezky[2], Ehud Fonio[3], Ofer Feinerman[3], Nir S. Gov[1]

1 Department of Chemical Physics, Weizmann Institute, Rehovot, Israel

2 Department of Biology, Emory University, Atlanta, Georgia, USA

3 Department of Complex Systems, Weizmann Institute, Rehovot, Israel

* Nir.Gov@Weizmann.ac.il

CONTENTS

1. Derivation of the analytical model 1
   a. The rate equation: Transition from the microscopic to the macroscopic view 1
   b. The equations of motion of the simplified model 3

2. Comparison to the tethered cargo model 4

3. Stability analysis derivations 5
   a. Linear stability 5
   b. Estimation of the homoclinic bifurcation 6

4. Relation between cargo size and individuality 7

5. Full 2d mathematical model 8

6. Discussion of the interactions of single ants with the obstacle edges 9

7. References 10

1. DERIVATION OF THE ANALYTICAL MODEL

a. The rate equation: Transition from the microscopic to the macroscopic view

The velocity of the cargo is given by a tug of war between the pullers on the front and back sides and is proportional to:

$$v \propto n_p^{front} - n_b^{back} \tag{S1}$$

By setting $n \equiv n_p^{front} - n_b^{back}$ and $N \equiv \frac{n_{tot}}{2}$ we can describe the system as a one step jump process where at any given epoch, the system at state n can jump forward or backward ($n \rightarrow n+1$ or

$n \to n-1$) with transition rates of α_n and β_n respectively within the sample space of $n \in [0, N]$.

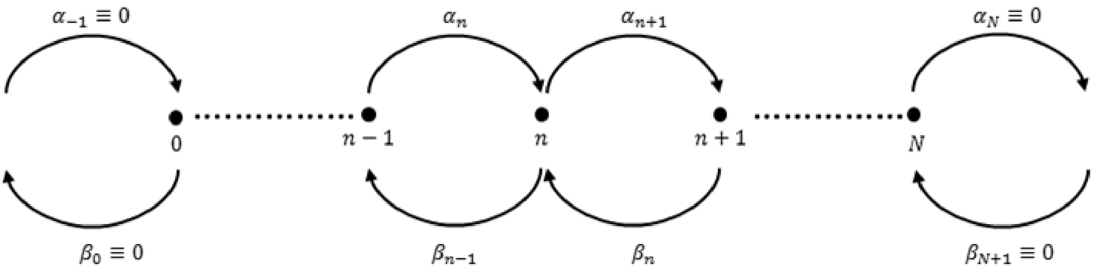

The gain term α_n is given by contributions from pullers at the back and lifters at the front:

$$\alpha_n = n_l^{front} r_{l\to p}^{front} + n_p^{back} r_{p\to l}^{back} = k_c \left(n_l^{front} + n_p^{back}\right) exp\left(\frac{f_{tot}}{F_{ind}}\right) \tag{S2}$$

and the loss term β_n is given by contributions from lifters at the front and pullers at the back:

$$\beta_n = n_p^{front} r_{p\to l}^{front} + n_l^{back} r_{l\to p}^{back} = k_c \left(n_l^{front} + n_p^{back}\right) exp\left(-\frac{f_{tot}}{F_{ind}}\right) \tag{S3}$$

The probability of finding the system in state n at time $t+dt$ is given by:

$$P_n(t+dt) = \alpha_{n-1}P_{n-1}(t)dt + \beta_{n+1}P_{n+1}(t)dt + (1 - \alpha_n dt + \beta_n dt)P_n(t) \tag{S4}$$

which yields the master equation for the given jump process:

$$\frac{dP_n(t)}{dt} = \alpha_{n-1}P_{n-1}(t) + \beta_{n+1}P_{n+1}(t) - (\alpha_n + \beta_n)P_n(t) \tag{S5}$$

By definition, the mean value of n can be written as:

$$\langle n(t)\rangle = \sum_{n=0}^{N} nP_n(t) \tag{S6}$$

and therefore the rate of change in $n(t)$ is given by:

$$\frac{d\langle n(t)\rangle}{dt} = \sum_{n=0}^{N} n\frac{dP_n(t)}{dt} \tag{S7}$$

Plugging in (S5) into (S6):

$$\frac{d\langle n(t)\rangle}{dt} = \underbrace{\sum_{n=0}^{N} n\alpha_{n-1}P_{n-1}(t)}_{n\to n+1} + \underbrace{\sum_{n=0}^{N} n\beta_{n+1}P_{n+1}(t)}_{n\to n-1} - \sum_{n=0}^{N} n(\alpha_n+\beta_n)P_n(t) \tag{S8}$$

$$= \underbrace{\sum_{n=-1}^{N-1} (n+1)\alpha_n P_n(t)}_{\alpha_{-1}\equiv 0\ ,\ \alpha_N \equiv 0} + \underbrace{\sum_{n=1}^{N+1} (n-1)\beta_n P_n(t)}_{\beta_0 \equiv 0\ ,\ \beta_{N+1}\equiv 0} - \sum_{n=0}^{N} n(\alpha_n+\beta_n)P_n(t) \tag{S9}$$

$$= \sum_{n=0}^{N} \left[(n+1)\alpha_n P_n(t) + (n-1)\beta_n P_n(t) - n(\alpha_n+\beta_n)P_n(t)\right] \tag{S10}$$

$$\Rightarrow \frac{d\langle n(t)\rangle}{dt} = \sum_{n=0}^{N} \alpha_n P_n(t) - \sum_{n=0}^{N} \beta_n P_n(t) \tag{S11}$$

Recognizing both terms on the r.h.s. of (S11) as the weighted averages of the gain and loss terms:

$$\frac{d\langle n(t)\rangle}{dt} = \langle \alpha_n \rangle - \langle \beta_n \rangle \tag{S12}$$

and by neglecting fluctuations around the mean we obtain a deterministic equation for the rate:

$$\frac{dn(t)}{dt} = \alpha_n - \beta_n \tag{S13}$$

where by plugging in (S2,S3) into (S13) we obtain the rate equation:

$$\frac{dn_p^{front}}{dt} - \frac{dn_p^{back}}{dt} = k_c \left[\left(n_l^{front} + n_p^{back}\right) exp\left(\frac{f_{tot}}{F_{ind}}\right) - \left(n_p^{front} + n_l^{back}\right) exp\left(-\frac{f_{tot}}{F_{ind}}\right) \right] \tag{S14}$$

b. The equations of motion of the simplified model

Starting by decomposing the force vector into an internal and external parts:

$$f_{tot} = f_{int} + f_{ext} \tag{S15}$$

The internal part will be the difference between the number of puller ants on both sides of the cargo lattice, and will also account the reduction of friction due to the lifter ants

$$f_{int} = \underbrace{f_0\left(n_p^{front} - n_p^{back}\right)}_{\text{pullers}} - \underbrace{h(f_s, n_l)}_{\text{lifters}} \tag{S16}$$

where

$$h(f_s, n_l) = f_s\left(1 - \Theta\left(\beta n_l - f_s\right) - \frac{\beta n_l}{f_s}\Theta\left(f_s - \beta n_l\right)\right) \tag{S17}$$

where Θ is the Heaviside step function, f_s is the static friction force due to the contact of the cargo with the surface, β is the magnitude of friction force reduced by a lifter ant, and n_l is the number of lifter ants.

Eq.S17 implies that each lifter ant reduces a factor of β from the static frictional force f_s, until enough lifters lifted the cargo above the surface ($\beta n_l > f_s$), such that $h(f_s, n_l) \to 0$. For simplicity, we consider throughout the model that the cargo is fully lifted above the surface, and neglect the contribution of the lifter ants.

The external part of the force balance S55 is given by:

$$f_{ext} = -f_0 G \cdot tanh\left(\frac{x}{\epsilon}\right) \tag{S18}$$

In the limit of $\epsilon \to 0$ ($tanh\left(\frac{x}{\epsilon}\right) \to sign(x)$), the informed ants act as a restoring force $\forall x$.

The c.o.m. velocity is given by normalizing f_{tot} by the cargo mechanical response coefficient γ:

$$v = \frac{f_0}{\gamma}\left(n_p^{front} - n_p^{back} - G \cdot tanh\left(\frac{x}{\epsilon}\right)\right) \tag{S19}$$

and therefore the acceleration is given by:

$$\frac{dv}{dt} = \underbrace{\frac{f_0}{\gamma}\left(\frac{dn_p^{front}}{dt} - \frac{dn_p^{back}}{dt}\right)}_{\text{internal}} - \underbrace{\frac{f_0}{\gamma}\frac{d}{dx}\left(G \cdot tanh\left(\frac{x}{\epsilon}\right)\right)\frac{dx}{dt}}_{\text{external}} \tag{S20}$$

By plugging in the rate equation (S14) into the acceleration (S20) we obtain:

$$\frac{dv}{dt} = \frac{k_c f_0}{\gamma}\left[\left(n_p^{front} + n_l^{back}\right) exp\left(\frac{f_{tot}}{F_{ind}}\right) - \left(n_p^{back} + n_l^{front}\right) exp\left(-\frac{f_{tot}}{F_{ind}}\right)\right] - \frac{v}{\epsilon}\frac{f_0 G}{\gamma} sech^2\left(\frac{x}{\epsilon}\right) \tag{S21}$$

For for a fully occupied lattice, the number of ants on both sides is given by:

$$n_p^{front} + n_l^{front} = \frac{n_{tot}}{2} \tag{S22}$$

$$n_p^{back} + n_l^{back} = \frac{n_{tot}}{2} \tag{S23}$$

and by adding and subtracting (S19) from (S22,S23) we obtain:

$$\frac{n_{tot}}{2} - \frac{f_{tot}}{f_0} - G \cdot tanh\left(\frac{x}{\epsilon}\right) = n_p^{front} + n_l^{back} \tag{S24}$$

$$\frac{n_{tot}}{2} + \frac{f_{tot}}{f_0} + G \cdot tanh\left(\frac{x}{\epsilon}\right) = n_p^{back} + n_l^{front} \tag{S25}$$

Therefore, by plugging in (S24,S25) into (S20) we obtain:

$$\frac{dv}{dt} = k_c\left[\frac{f_0 n_{tot}}{\gamma} sinh\left(\frac{f_{tot}/\gamma}{F_{ind}/\gamma}\right) - 2\left(\frac{f_{tot}}{\gamma} + \frac{f_0 G}{\gamma} tanh\left(\frac{x}{\epsilon}\right)\right) cosh\left(\frac{f_{tot}/\gamma}{F_{ind}/\gamma}\right)\right] - \frac{v}{\epsilon}\frac{f_0 G}{\gamma} sech^2\left(\frac{x}{\epsilon}\right) \tag{S26}$$

$$= k_c\left[n \cdot sinh\left(\frac{v}{f_{ind}}\right) - 2\left(v + g \cdot tanh\left(\frac{x}{\epsilon}\right)\right) cosh\left(\frac{v}{f_{ind}}\right)\right] - g\frac{v}{\epsilon} sech^2\left(\frac{x}{\epsilon}\right) \tag{S27}$$

where in (S27) we rescale the parameters with force units by γ: $n = \frac{f_0 n_{tot}}{\gamma}$, $v = \frac{f_{tot}}{\gamma}$, $f_{ind} = \frac{F_{ind}}{\gamma}$, $g = \frac{f_0 G}{\gamma}$, such that all terms have units of $[L][T^{-2}]$.

2. COMPARISON TO THE TETHERED CARGO MODEL

In [1] the dynamics of a cargo that is constrained by a tether was investigated. It was found that the cargo is pulled in the direction of the nest until the tether is taught, and then begins to perform oscillations. Unlike our system, the informed ants pull the cargo in a direction that is mostly orthogonal to the allowed motion. The equations of motion for the both models are given by:

$$\text{tethered cargo}\begin{cases}\dot{\theta} &= \frac{1}{L}v \\ \dot{v} &= k_c\left[n \cdot sinh\left(\frac{v}{f_{ind}}\right) - 2\left(v + \boldsymbol{g \cdot sin(\theta)}\right) cosh\left(\frac{v}{f_{ind}}\right)\right] \boldsymbol{- v\frac{g}{L} cos(\theta)}\end{cases} \tag{S28}$$

$$\text{rigid obstacle}\begin{cases}\dot{x} &= v \\ \dot{v} &= k_c\left[n \cdot sinh\left(\frac{v}{f_{ind}}\right) - 2\left(v + \boldsymbol{g \cdot tanh\left(\frac{x}{\epsilon}\right)}\right) cosh\left(\frac{v}{f_{ind}}\right)\right] \boldsymbol{- v\frac{g}{\epsilon} sech^2\left(\frac{x}{\epsilon}\right)}\end{cases} \tag{S29}$$

The difference between the two models is the external force and its derivative (the emboldened terms). While for the rigid obstacle case the restoring force is localized to $x = 0$, in the tethered cargo case it is dependent on the angle θ, and therefore effectively directed (in a non uniform manner) to a scent trail with width of $2Lsin\theta_{max}$, where L is the tether length and θ_{max} is the maximal aperture the tethered cargo can take with respect to the origin. Comparison between the solution of both models shows that when $\epsilon \geq 0.5$ both models coincide (Fig.A) at a parameter range where there is no bi-stability.

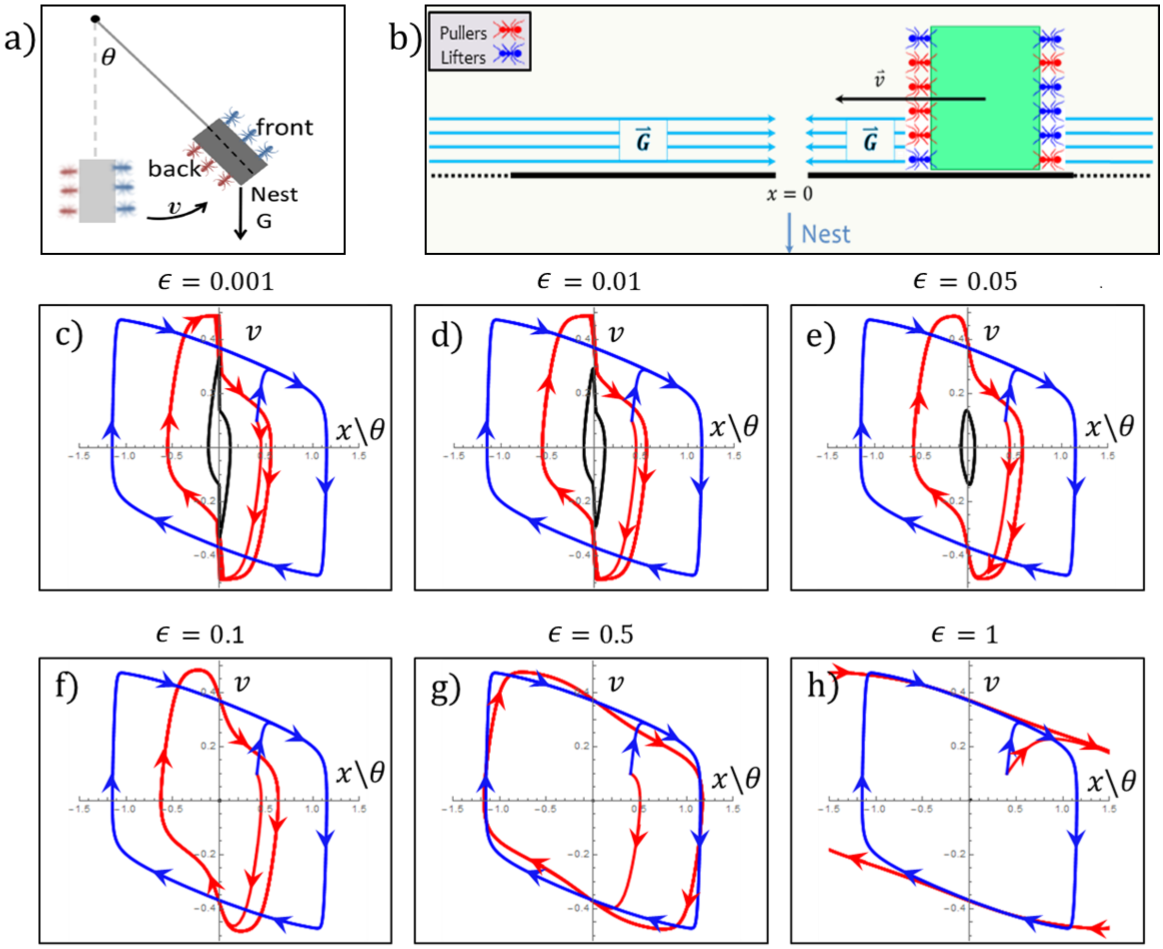

FIG. A. Comparison between the tethered cargo and linear obstacle models. (a) The tethered cargo model. (b) The linear obstacle model. (c-h) An overlay of the solutions of both models using the default parameters and $L = 5$ [cm]. Blue - tethered cargo. Red - rigid obstacle. Black - separatrix (linear obstacle solution).

3. STABILITY ANALYSIS DERIVATIONS

a. Linear stability

The dynamical system is given by:

$$\dot{x} = v \tag{S30}$$

$$\dot{v} = k_c \left[n \cdot sinh\left(\frac{v}{f_{ind}}\right) - 2\left(v + g \cdot tanh\left(\frac{x}{\epsilon}\right)\right) cosh\left(\frac{v}{f_{ind}}\right)\right] - v\frac{g}{\epsilon}sech^2\left(\frac{x}{\epsilon}\right) \tag{S31}$$

Finding the fixed points:

$$\dot{x} = 0 \longrightarrow v^* = 0 \tag{S32}$$

$$\dot{v} = 0 \longrightarrow -2g \cdot tanh\left(\frac{x}{\epsilon}\right) = 0 \longrightarrow x^* = 0 \tag{S33}$$

Linearizing (S30) and (S31) around the fixed point $(x^*, v^*) = (0, 0)$:

$$\dot{x} = x \tag{S34}$$

$$\dot{v} \approx \frac{-2g}{\epsilon}x + \left(k_c\left(\frac{n}{f_{ind}} - 2\right) - \frac{g}{\epsilon}\right)v \tag{S35}$$

The eigenvalues of (S34,S35) are given by:

$$\lambda_{1,2} = \frac{1}{2}\left(\mu \pm \sqrt{\mu^2 - 4\omega^2}\right) \tag{S36}$$

where:

$$\mu = k_c\left(\frac{n}{f_{ind}} - 2\right) - \frac{g}{\epsilon} \tag{S37}$$

$$\omega = \sqrt{\frac{2g}{\epsilon}} \tag{S38}$$

The fixed point will change stability when $\mu = 0$, which corresponds to the point where $Re[\lambda] = 0$. Therefore, the critical f_{ind} for the Hopf bifurcation is given by:

$$\mu = 0 \longrightarrow f_c^{(1)} = \frac{n}{2}\left(\frac{1}{1 + \frac{g}{2k_c\epsilon}}\right) \tag{S39}$$

b. Estimation of the homoclinic bifurcation

The acceleration of system is given by:

$$q(v, x) = k_c\left[n \cdot sinh\left(\frac{v}{f_{ind}}\right) - 2\left(v + g \cdot tanh\left(\frac{x}{\epsilon}\right)\right)cosh\left(\frac{v}{f_{ind}}\right)\right] - v\frac{g}{\epsilon}sech^2\left(\frac{x}{\epsilon}\right) \tag{S40}$$

At the transition between phases (i) and (ii) two nullclines merge, and the free motion changes to relaxation oscillations [2] .The transition point is an extrema of (S40).
By treating the system separately for each half space of x ($tanh\left(\frac{x}{\epsilon}\right) \rightarrow sign(x)$) we obtain:

$$q^{\pm}(v) = 0 \rightarrow tanh\left(\frac{v}{f_{ind}}\right) = \frac{v \mp g}{\frac{n}{2}} \tag{S41}$$

$$\frac{dq^{\pm}(v)}{dv} = 0 \rightarrow tanh\left(\frac{v}{f_{ind}}\right) = \frac{\frac{n}{2} - f_{ind}}{v \pm g} \tag{S42}$$

and by equating (S41) and (S42) we obtain the velocity at the transition:

$$v^{\pm} = \pm g \mp \sqrt{\frac{n}{2}\left(\frac{n}{2} - f_{ind}\right)} \tag{S43}$$

The approximation of the critical point of bifurcation is given by pluggin in (S43) into (S41):

$$f_c^{(2)} = \frac{n}{2}sech^2\left(\frac{-g + \sqrt{\frac{n}{2}\left(\frac{n}{2} - f_c^{(2)}\right)}}{f_c^{(2)}}\right) \tag{S44}$$

4. RELATION BETWEEN CARGO SIZE AND INDIVIDUALITY

The number of ants N that can occupy a circular cargo with radius R is given by

$$N \approx \frac{2\pi R}{L} \tag{S45}$$

where L is the average width of an ant, and R is the radius of the circular cargo.
The number of pullers that occupy the cargo can be written as

$$N_p = bN \tag{S46}$$

where b is a fraction. By Plugging Eq.S46 into Eq.S45, we obtain that the number of pullers that are attached to the cargo, is proportional to the cargo size

$$N_p \sim R \tag{S47}$$

The role switching rate is given by

$$r_{l\leftrightarrow p} = k_c exp\left(\pm\frac{\vec{f}_{tot}\cdot\vec{p}_i}{F_{ind}}\right) \tag{S48}$$

where $\vec{f}_{tot}$ is the total applied force, $\vec{p}_i$ is the body axis unit vector of an individual ant labeled by i, and F_{ind} is the individuality parameter.
When the cargo is in a persistent motion, the total force f_{tot} can be approximated by

$$\vec{f}_{tot} = f_0 N_p \hat{f}_{tot} \tag{S49}$$

and the body axis unit vector of each ant can be approximated by

$$\vec{p}_i = \hat{f}_{tot} \quad \forall i \tag{S50}$$

where f_0 is the magnitude of force applied by a single puller. What Eqs. S49,S50 imply, is that the total force is given by the puller ants, that pull the cargo in a single persistent direction.
By plugging Eq.S49,S50 into Eq.S48 we obtain

$$r_{l\leftrightarrow p} = k_c exp\left(\pm\frac{f_0 N_p \hat{f}_{tot}\cdot\hat{f}_{tot}}{F_{ind}}\right) \tag{S51}$$

Therefore

$$log\left(r_{l\to p}\right) \approx \frac{N_p}{F_{ind}} \tag{S52}$$

And by using the relation between the number of pullers and the cargo size (Eq.S47) we obtain

$$log\left(r_{l\to p}\right) \approx \frac{R}{F_{ind}} \tag{S53}$$

Therefore an increase in R is equivalent to an effective decrease of F_{ind}, and a decrease in R is equivalent to an effective increase of F_{ind}. For the circular cargoes we use in the experiments, we have a linear relation between the mass and the radius. i.e $m \sim R$ (Fig.B).

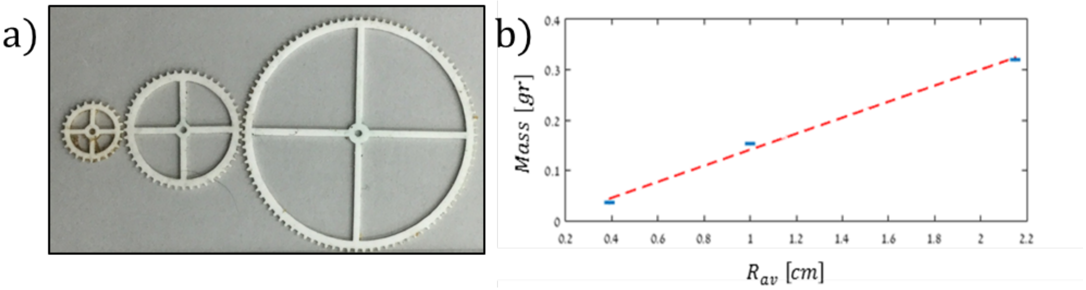

FIG. B. (a) The three cargoes used in the experiments ($R = 0.5, 1, 2$ [cm]). (b) Cargo mass vs. Cargo radius (average of the inner and outer rings radii).

5. FULL 2D MATHEMATICAL MODEL

For a full 2D description we model a ring-like cargo as a circular lattice with n_{max} sites (Fig.Ca) [3]. Each site i can be occupied by a puller, a lifter, or remain empty and is denoted by:

$$\alpha_i = \begin{cases} 1 & \text{puller ant} \\ -1 & \text{lifter ant} \\ 0 & \text{empty site} \end{cases} \tag{S54}$$

The total force and c.o.m. velocity are given by:

$$\vec{f}_{tot} = f_0 \sum_{i=1}^{n_{max}} \left(\frac{\alpha_i + 1}{2}\right) \vec{p}_i \tag{S55}$$

$$\vec{v} = \frac{1}{\gamma}\left(\vec{f}_{tot} - (f_s - f_k)\hat{f}_{tot}\right) \tag{S56}$$

where the force f_0 exerted by a single puller is considered equal for all the ants, and $\vec{p}_i$ is the body axis unit vector of an ant on site i:

$$\vec{p}_i = cos(\theta_i + \phi_i)\hat{x} + sin(\theta_i + \phi_i)\hat{y} \tag{S57}$$

where θ_i is the angle of site i around the cargo with respect to the chosen frame of reference, and ϕ_i is the local orientation of the ant with respect to θ_i.

The static friction is parameterized by the coefficient is given by f_s, which is reduced with respect to the lifter activity by:

$$\tilde{f}_k = -f_0\beta \sum_{i=1}^{n_{max}} \left(\tfrac{\alpha_i - 1}{2}\right)$$
$$f_k = \begin{cases} \tilde{f}_k & \tilde{f}_k < f_s \\ f_s & \tilde{f}_k > f_s \end{cases} \tag{S58}$$

where βf_0 is the reduction in friction force by a single lifter, and is equal for all sites of a cargo with radial symmetry. The angular velocity of the cargo is given by:

$$\vec{\omega} = \frac{1}{\gamma_{rot}}\vec{\tau}_{tot} = \frac{1}{\gamma_{rot}}\left(f_0 \sum_{i=1}^{n_{max}} \left(\frac{\alpha_i + 1}{2}\right) sin(\phi_i) - \vec{\tau}_{kin}\right) \tag{S59}$$

where γ_{rot} is the cargo rotation response coefficient and $\vec{\tau}_{kin}$ is the kinetic torque friction acting in the same manner as (S58).

The dynamics are stochastic and simulated using a Gillespie algorithm [4], where ants can attach, detach, role switch, reorient (change its angle ϕ_i) and forget (transition from informed to uninformed). The general dynamic scheme of the 2D model is described in Fig.Ca. Qualitative realizations of the dynamics in an ordered and disordered phases in a free motion (no obstacles) are shown in Fig.Cb,c.

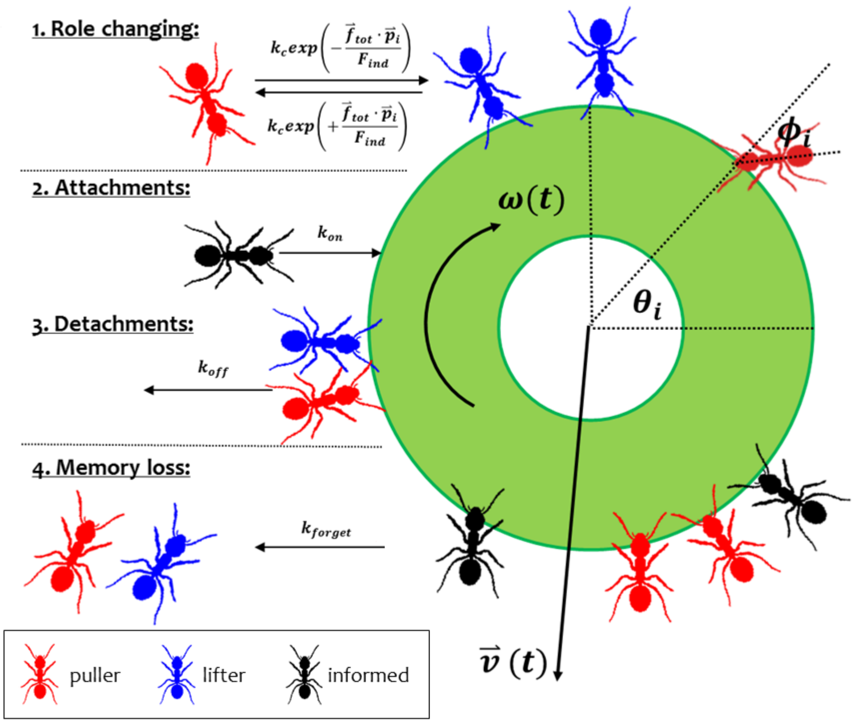

FIG. C. Representation of the two dimensional mathematical model of cooperative transport by ants. $v(t)$ is the velocity of the cargo center of mass, $\omega(t)$ is the orientation angular velocity of the cargo. θ_i is the angle of an attached ant labeled by i with respect to the chosen lab frame of reference, ϕ_i is the orientation of a single ant with respect to θ_i, and (1-4) indicate the stochastic processes of role changing, attachments, detachments and loss of memory by informed ants.

6. DISCUSSION OF THE INTERACTIONS OF SINGLE ANTS WITH THE OBSTACLE EDGES

In the experimental setup we used, the cargo did not reach the side edges. Although we attribute this fact to the informed ants that pull towards the hole [5], ants were free to forage everywhere inside the obstacle. Since according to our model single ants can hold information that can be valuable to the group, we ask if single ants that arrived to the side edges, can act as "informed" and take the cargo away from these edges. In Fig.D we provide an example from an analysis that counts the number of ants near the obstacle edges and inside the area of oscillations where the cargo moved.

The analysis shows that the number of ants near the edges is much smaller compared to the number of ants along the rest of the obstacle. Throughout all the experiments that we have conducted, we have not found any case where a single ant that reached the obstacle edges, changed its direction of motion towards the cargo and attached to it.

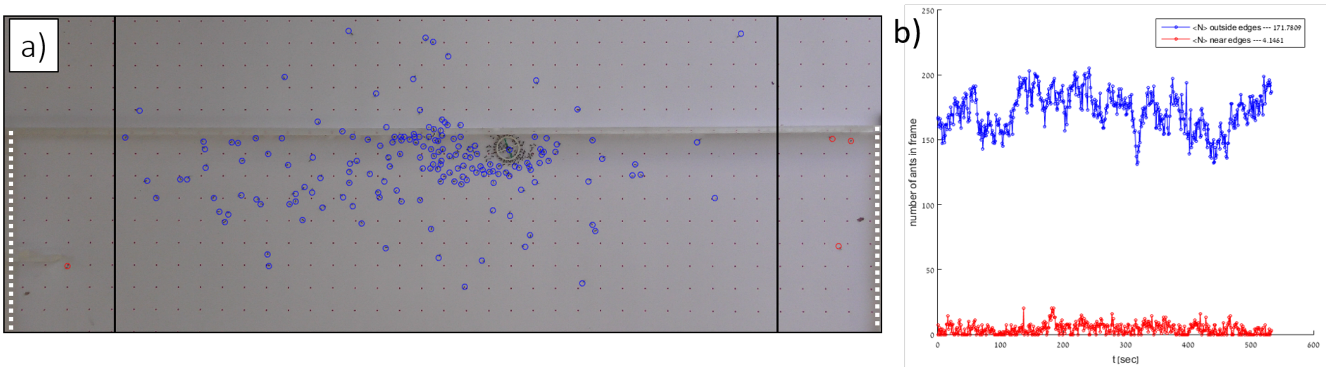

FIG. D. (a) A snapshot from an experiment showing the number of ants near the side edges (red circles) and in along the rest of the frame (blue circles). Black lines indicate the spatial extent of the regions chosen to distinguish between the two populations. Dashed white lines indicate the obstacle's side edges. (b) A time series from $\sim$ 10 minutes of experimental data, showing the number of ants counted near the edges (red), and the number of ants counted along the rest of the frame (blue). The calculation is done with respect to the areas indicated in section (a) of the figure.

7. REFERENCES

[1] A. Gelblum, I. Pinkoviezky, E. Fonio, N. S. Gov, and O. Feinerman, Proceedings of the National Academy of Sciences **113**, 14615 (2016).

[2] S. H. Strogatz, *Nonlinear dynamics and chaos: with applications to physics, biology, chemistry, and engineering* (Westview press, 2014).

[3] A. Gelblum, I. Pinkoviezky, E. Fonio, A. Ghosh, N. Gov, and O. Feinerman, Nature communications **6** (2015).

[4] D. T. Gillespie, Journal of computational physics **22**, 403 (1976).

[5] E. Fonio, Y. Heyman, L. Boczkowski, A. Gelblum, A. Kosowski, A. Korman, and O. Feinerman, eLife **5**, e20185 (2016).
