# Supplementary figures and images for: Bi-stability in cooperative transport by ants in the presence of obstacles
Source: PLoS Comput Biol. 2018 May 10;14(5):e1006068. doi: 10.1371/journal.pcbi.1006068 (PMC5944914; doi:10.1371/journal.pcbi.1006068)

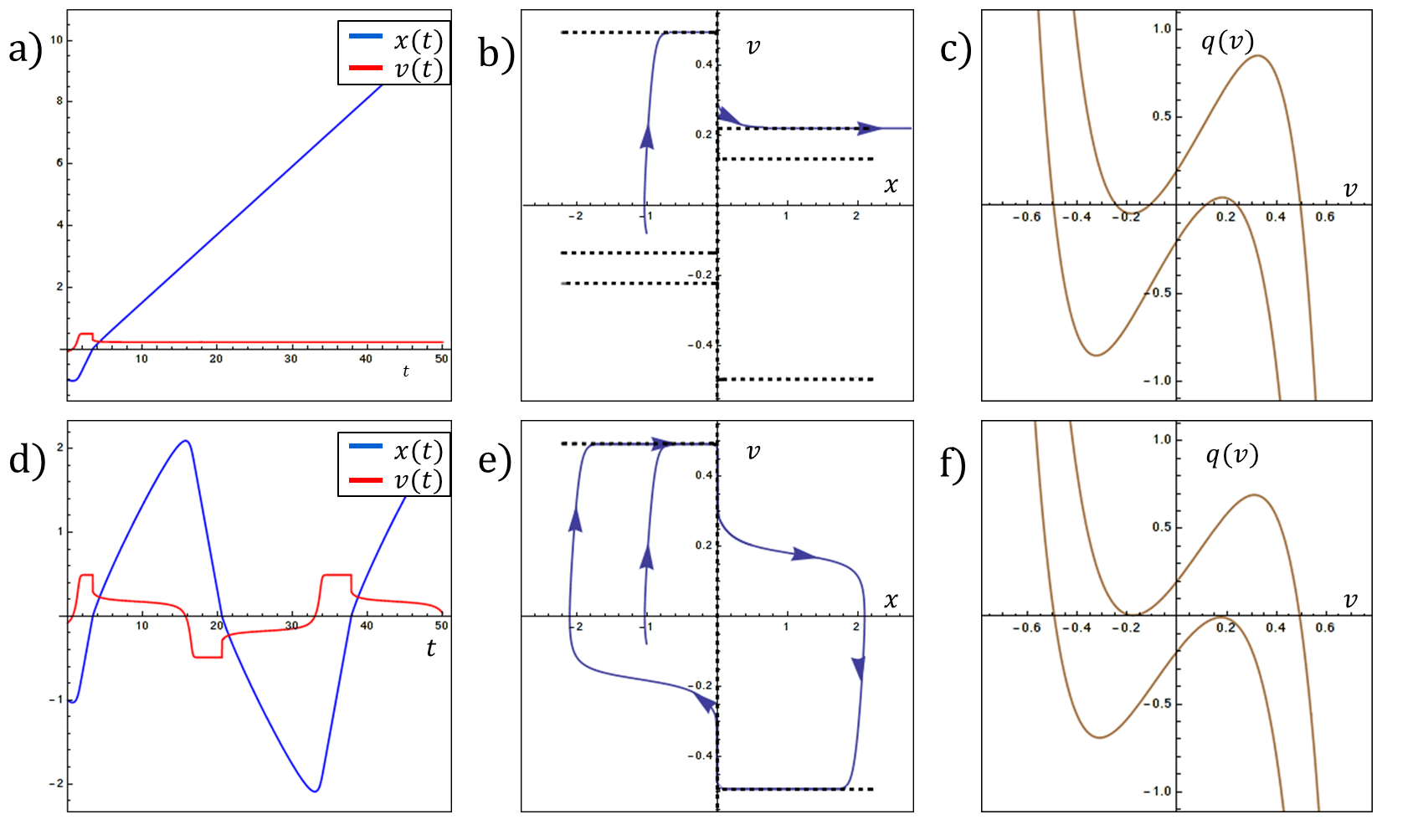

Supplement: S1 Fig — Before the transition (find = 0.18): (a) Position and velocity as a function of time. (b) Phase space trajectories (blue) and nullclines (dashed black). (c) The solution of q(v, x) for each half space of x under the approximation of ϵ → 0. After the transition (find = 0.21): (d) Position and velocity as a function of time. (e) Phase space trajectory (blue) and nullclines (dashed black). (f) The solution of q(v, x) for each half space of x under the approximation of ϵ → 0. (TIF) [file pcbi.1006068.s002.tif]

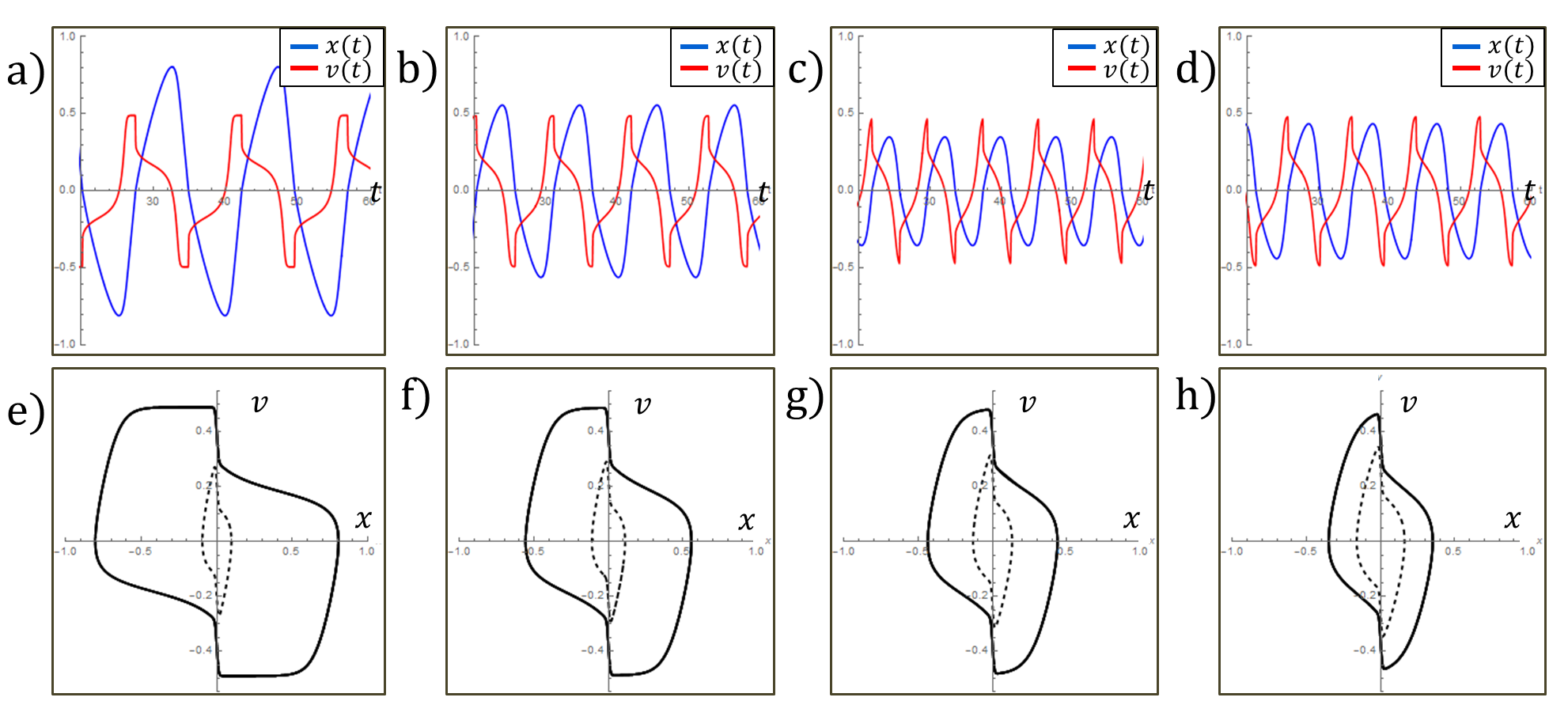

Supplement: S2 Fig — Solutions of the deterministic model along the oscillatory bi-stable region for parameters g = 0.1 and find = 0.22, 0.23, 0.24, 0.25. (a-d) Position and velocity time series, blue curve is the position, red curve is the velocity. (e-h) Phase space trajectories, solid line is the stable limit cycle, dashed line is the unstable limit cycle (separatrix). (TIF) [file pcbi.1006068.s003.tif]
